# Clinical evidence and potential mechanisms of traditional Chinese medicine for refractory heart failure: a literature review and perspectives

**DOI:** 10.3389/fcvm.2024.1369642

**Published:** 2024-04-23

**Authors:** Liuli Guo, Zhihua Yang, Wenshuai Feng, Yiman Liu, Zhenzhen Li, Pengwei Zhuang, Ming Ren

**Affiliations:** ^1^ Baokang Hospital Affiliated to Tianjin University of Traditional Chinese Medicine Tianjin, China; ^2^ Institute of Traditional Chinese Medicine, Tianjin University of Traditional Chinese Medicine, Tianjin, China; ^3^ First Teaching Hospital of Tianjin University of Traditional Chinese Medicine, Tianjin, China

**Keywords:** refractory heart failure, traditional Chinese medicine, clinical evidence, potential mechanisms, inflammatory response, vascular endothelial function, oxidative stress

## Abstract

Refractory heart failure (RHF), or end-stage heart failure, has a poor prognosis and high case fatality rate, making it one of the therapeutic difficulties in the cardiovascular field. Despite the continuous abundance of methods and means for treating RHF in modern medicine, it still cannot meet the clinical needs of patients with RHF. How to further reduce the mortality rate and readmission rate of patients with RHF and improve their quality of life is still a difficult point in current research. In China, traditional Chinese medicine (TCM) has been widely used and has accumulated rich experience in the treatment of RHF due to its unique efficacy and safety advantages. Based on this, we comprehensively summarized and analyzed the clinical evidence and mechanism of action of TCM in the treatment of RHF and proposed urgent scientific issues and future research strategies for the treatment of RHF with TCM, to provide reference for the treatment of RHF.

## 1 Introduction

Refractory heart failure (RHF), also known as intractable heart failure or end-stage heart failure, refers to a group of clinical syndromes in which patients, after standardized management and treatment, remain unable to be effectively controlled or deteriorate even further; their condition is recurrent, requires repeated hospitalization, and has a high mortality rate (1, 2). In recent years, the prevalence of heart failure has continuously increased, and the number of patients with RHF has also increased. Epidemiology shows that the number of people with heart failure has reached as high as 64.3 million worldwide (3), of which RHF accounts for approximately 6%–25% (4–6). Currently, although advanced means, such as implantation of ventricular assist devices, surgical ventricular remodeling, and cardiac transplantation, are available for the treatment of RHF, medication is still the main treatment modality (7). The main drugs used in the clinical treatment of RHF are diuretics, inotropic agents, vasodilators, and neuroendocrine inhibitors. Even though these drugs can effectively improve patients’ clinical symptoms, long-term use can lead to adverse reactions and even worsen heart failure, exacerbating the condition (8). Therefore, how to find new targets for the treatment of heart failure, prevent and delay myocardial remodeling and then reduce the rehospitalization rate and mortality of patients with RHF, and improve the quality of survival is still the hotspot and difficult point in the field of cardiovascular research at present.

In recent years, the traditional Chinese medicine (TCM) treatment for RHF has increasingly been reported. Clinical studies have shown that the combined use of TCM based on the conventional treatment of Western medicine is superior to the treatment of Western medicine alone in improving clinical symptoms, such as chest tightness and shortness of breath, fatigue, dyspnea, and urine oligoedema, in patients with RHF, improving exercise tolerance and improving long-term outcomes. In this article, we systematically summarize the clinical evidence and the mechanism of action of TCM for the treatment of RHF, as well as the scientific problems that exist in TCM for the treatment of RHF, to provide a reference for the clinical treatment of RHF.

## 2 TCM in the treatment of RHF

### 2.1 Understanding RHF in TCM theory

There is no specific record of RHF in ancient Chinese medicine, and according to its clinical manifestations, it can be classified as “palpitation,” “severe palpitation,” “asthma,” “edema,” and other categories. At present, there is a lack of systematic research on the characteristics of TCM syndromes in RHF, and clinical physicians tend to equate it with heart failure. Although RHF belongs to HF, it is in the end stage of the disease, and the treatment is more difficult. The characteristics of RHF syndrome are indeed different from those of heart failure. There is no unified understanding of the etiology, pathogenesis, and characteristics of this disease in TCM. Based on the literature, some scholars classify the TCM characteristics of RHF as a deficiency in origin and excess in superficiality, and the combination of deficiency and excess. The deficiency in origin is mainly Yang deficiency, and the excess in superficiality is mainly blood stasis and water retention. Some scholars believe that the main pathogenesis of RHF is heart-kidney Yang deficiency and the combination of blood stasis and water (9). The principle of treatment should balance the priorities, tonify deficiency, and reduce excess. The first step in treatment is to support the Yang deficiency and consolidate the foundation, dissipate blood stasis, and promote diuresis, while also regulating qi and dispersing stasis, resolving phlegm and removing blood stasis, and capturing and promoting drinking (10).

### 2.2 Clinical evidence of TCM for RHF

**Figure 1:**
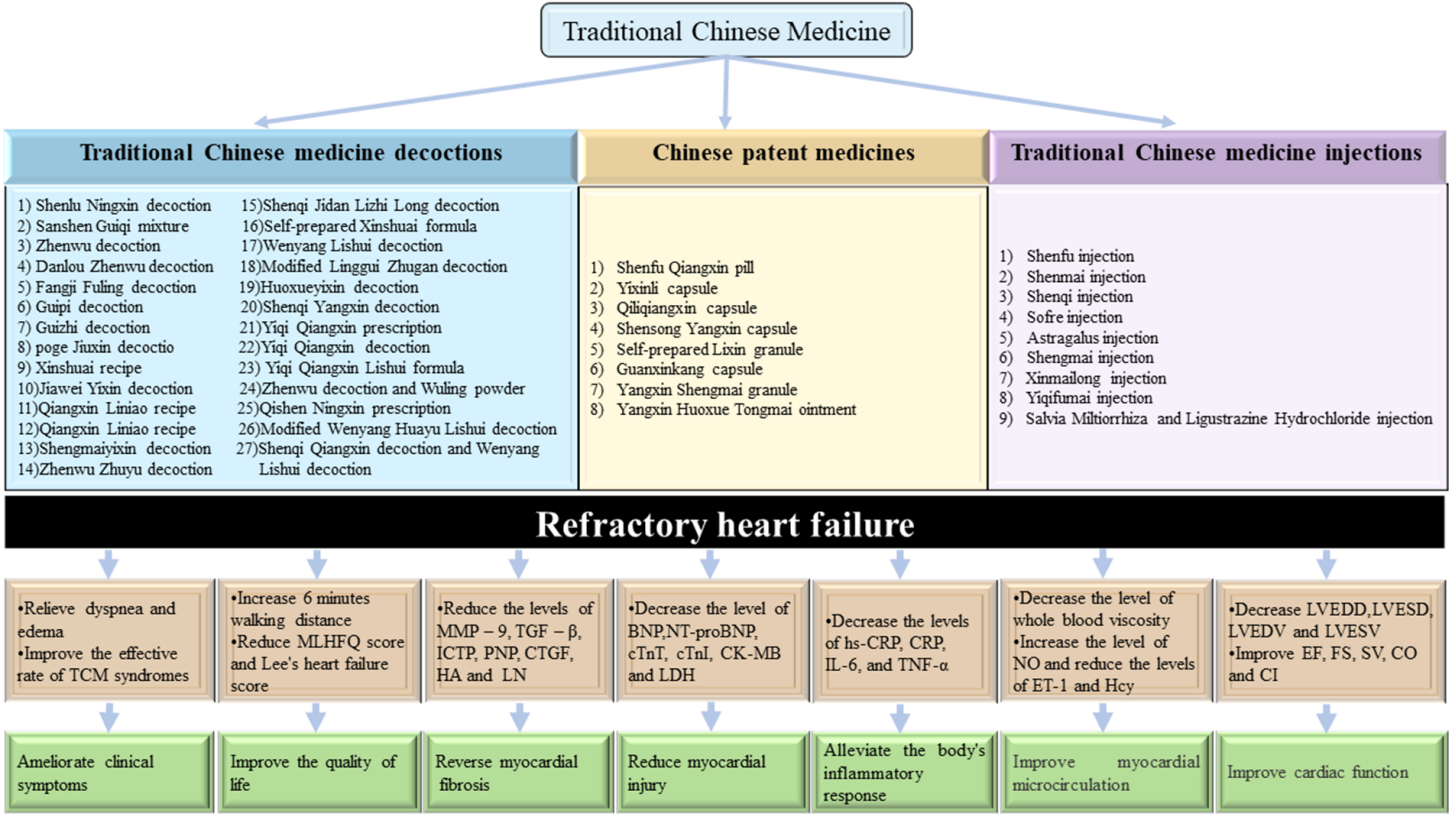
Clinical evidence of TCM for RHF.

This article summarizes 58 representative randomized controlled trials (RCTs), including 28 TCM decoctions, 12 Chinese patent medicines (CPMs), and 18 TCM injections. According to the available clinical data, there have been many clinical trials on the clinical advantages of TCM in the prevention and treatment of RHF, as shown in Figure 1. Supplementary Table S1 summarizes the clinical evidence for the TCM treatment of RHF, including (1) clinical manifestations, (2) cardiac function indexes, and (3) laboratory results.

#### 2.2.1 TCM decoctions for RHF treatment

Clinical symptoms
  (1) Shenlu Ningxin decoction (11), modified Linggui Zhugan decoction (12), Shenqi Qiangxin decoction and Wenyang Lishui decoction (13), Danlou Zhenwu decoction (14), modified Wenyang Huayu Lishui decoction (15), Zhenwu Zhuyu decoction (16), Yiqi Qiangxin Lishui formula (17), Jiawei Yixin decoction (18), and Wenyang Lishui decoction (19) can all improve symptoms of palpitations, chest tightness, wheezing, shortness of breath, and fatigue in patients. Shenlu Ningxin decoction (11), self-prepared Xinshuai formula (20), and Yiqi Qiangxin prescription (21) can improve symptoms such as palpitations, bloating, suffocation, cyanosis of the lips, and chills in the limbs of patients. Shenqi Qiangxin decoction and Wenyang Lishui decoction (13) and Yiqi Qiangxin Lishui formula (17) can improve symptoms such as difficulty breathing, poor appetite, and urinary edema in patients. Yiqi Qiangxin decoction (22), modified Wenyang Huayu Lishui decoction (15), and Zhenwu Zhuyu decoction (23) can all slow down heart rate; Poge Jiuxin decoction (24) and self-prepared Xinshuai formula (20) can reduce Lee's heart failure score. Shenlu Ningxin decoction (11), Jiawei Yixin decoction (18), and Yiqi Qiangxin prescription (21) reduce the Minnesota Living with Heart Failure Questionnaire (MLHFQ) and improve patients’ quality of life. Zhenwu Zhuyu decoction (16), Shenqi Yangxin decoction (25), and Danlou Zhenwu decoction (14) can increase walking distance by 6 min and improve exercise tolerance.
Echocardiography
  (2) Shenlu Ningxin decoction (11), Shenqi Jidan Lizhi Long decoction (26), Shenqi Qiangxin decoction and Wenyang Lishui decoction (13), Danlou Zhenwu decoction (14), Guipi decoction (27), modified Wenyang Huayu Lishui decoction (15), modified Linggui Zhugan decoction (12), Jiawei Yixin decoction (18), Wenyang Lishui decoction (19), Fangji Fuling decoction (28), Shenqi Yangxin decoction (25), Yiqi Qiangxin prescription (21), Dingchuan decoction (29), and Qishen Ningxin prescription (30) can improve heart function, reduce left ventricular end diastolic diameter (LVEDD), and left ventricular end-systolic diameter (LVESD), reduce left ventricular end-systolic volume (LVESV) and increase left ventricular ejection fraction (LVEF). Shenqi Jidan Lizhi Long decoction (26), Jiawei Yixin decoction (18), Poge Jiuxin decoction (24), and Yiqi Qiangxin decoction (22) increase the heart index (CI). Guizhi decoction (31), Zhenwu Zhuyu decoction (16), and Yiqi Qiangxin prescription (21) increase cardiac output (CO). Shenlu Ningxin decoction (11), Danlou Zhenwu decoction (14), Guizhi decoction (31), Zhenwu Zhuyu decoction (16), and Zhenwu decoction (32) increase stroke volume (SV).
Biochemical indicators
  (3) Shenlu Ningxin decoction (11), Danlou Zhenwu decoction (14), Guipi decoction (27), Guizhi decoction (31), Jiawei Yixin decoction (18), Shengmaiyixin decoction (33), Sanshen Guiqi mixture (34), Zhenwu Zhuyu decoction (23), Shenqi Yangxin decoction (25), modified Wenyang Huayu Lishui decoction (15), Poge Jiuxin decoction (24), Wenyang Lishui decoction (19), Xinshuai recipe (35), Yiqi Qiangxin Lishui formula (17), and Dingchuan decoction (29) can significantly reduce N-terminal pro-B-type natriuretic peptide (NT-proBNP) or brain natriuretic peptide (BNP). Zhenwu Zhuyu Decoction (23) can significantly reduce inflammatory factors, such as tumor necrosis factor-α (TNF-α), interleukin-6 (IL-6), and C-reactive protein (CRP). Shenlu Ningxin Decoction (11) reduced the levels of transforming growth factor-β1 (TGF-β1) and matrix metalloproteinase-9 (MMP-9). Guizhi decoction (31) can increase 24 h urine volume and serum sodium and reduce serum creatinine. Qiangxin Liniao recipe (36) can accelerate the improvement of symptoms such as hypotension, renal insufficiency, and insufficient blood volume. Zhenwu Zhuyu Decoction (23), Huoxueyixin decoction (37), Zhenwu decoction, and Wuling powder (38) can reduce the levels of whole blood low shear viscosity, whole blood high shear viscosity, fibrinogen, and plasma viscosity.

#### 2.2.2 TCM patent medicines for RHF treatment

Clinical symptoms
  (1) The Qiliqiangxin capsule (39), Yangxin Huoxue Tongmai ointment (40), Yixinli capsule (41), and self-prepared Lixin granule (42) improved the condition of patients with RHF and reduced cardiovascular dysfunctions. The most typical clinical symptoms include palpitations, shortness of breath and chest tightness, wheezing, fatigue, cough and expectoration, and edema. Self-prepared Lixin granule (42) improved the symptoms of jugular vein distension and bilateral lung moist rales. The Yixinli capsule (41), Yangxin Shengmai granule (43), and Qiliqiangxin capsule (44) can increase the 6-min walking distance (6MWD) and improve exercise tolerance. In addition, the Qiliqiangxin capsule (45) can improve the quality of life of patients and reduce the readmission rate.
Echocardiography
  (2) The Shenfu Qiangxin pill (46, 47), Qiliqiangxin capsule (45), Yangxin Huoxue Tongmai ointment (40), Yixinli capsule (41), Shensong Yangxin capsule (48), Guanxinkang capsule (49), and self-prepared Lixin granule (42) can increase LVEF and reduce LVESD, LVEDD, LVESV, and left ventricular end diastolic volume (LVEDV). The Qiliqiangxin capsule (39) can increase CI, SV, myocardial contractility, and venous return blood volume. The Yixinli capsule (41) and Yangxin Huoxue Tongmai ointment (40) improve the grading evaluation of heart function.
Biochemical indicators
  (3) The Shenfu Qiangxin pill (46), Qiliqiangxin capsule (39), and Shensong Yangxin capsule (48) can significantly reduce the level of serum NT-proBNP. The Shenfu Qiangxin pill (46) and Shensong Yangxin capsule (48) can reduce the levels of serum cTnT and cTnI. The Shenfu Qiangxin pill (47) reduces the serum levels of heart-fatty acid binding protein (H-FABP), creatine kinase myocardial bound (CK-MB), procollagen type I carboxy terminal peptide (ICTP), type III procollagen amino terminal peptide (PIIINP), connective tissue growth factor (CTGF), hyaluronic acid (HA), and laminin (LN) in patients. The Qiliqiangxin capsule (39, 50) can increase total cholesterol (TC), triglyceride (TG), low-density lipoprotein cholesterol (LDL-C) levels, reduce high density lipoprotein (HDL-C) indicators, and regulate blood lipid levels. In addition, the Shensong Yangxin capsule (48) can reduce the levels of inflammatory mediator hypersensitive C-reactive protein (hs-CRP).

#### 2.2.3 TCM injections for RHF treatment

Clinical symptoms
  (1) Shenfu injection (51), Astragalus injection (52), Shengmai injection (53), Xinmailong injection (54), and Sofre injection (55) can improve the clinical total effective rate, and improve the clinical symptoms, such as shortness of breath and fatigue, chest tightness and suffocation, tachypnea, palpitation, lower limb edema, and so on. Shengmai injection (56) can alleviate symptoms such as systemic fatigue, lower limb edema, and liver enlargement, as well as alleviate symptoms of total heart failure, such as jugular vein dilation and purple forceps. Shenfu injection (51), Shenmai injection (57), Sofre Injection (55), Xinmailong injection (58), and Yiqifumai injection (59) can increase the 6-min walking distance of patients and improve exercise endurance. Astragalus injection (52) and Sofre injection (55) can shorten hospitalization time and reduce the rate of readmission. Shenfu injection (51) and Xinmailong injection (60) can improve TCM syndrome scores and Minnesota Heart Failure Quality of Life Questionnaire scores, and improve quality of life.
Echocardiography
  (2) Shenfu injection (61–63), Shenmai injection (57, 64), Astragalus injection (65), Sofre Injection (55), and Xinmailong injection (54) improve cardiac function, reduce LVEDD, LVEDV, LVESD, and LVESV levels, increase fraction shortening (FS) levels, and increase LVEF. Shenfu injection (61, 62), Shenmai injection (64, 66), Sofre injection (55), and Salviae Miltiorrhizae and Ligustrazine Hydrochloride injection (67) can increase SV. Shenfu injection (63) and Shenmai injection (57, 64) increase the output per minute (CO). Shenmai injection (57, 64) and Sofre injection (55) increased the CI level.
Biochemical indicators
  (3) Shenfu injection (51, 61), Shenqi injection (68), Astragalus injection (65), Salviae Miltiorrhizae and Ligustrazine Hydrochloride injection (67), Xinmailong injection (58, 69), and Yiqifumai injection (59) can reduce the levels of NT-proBNP or BNP. Shenfu injection (61–63) can reduce the levels of inflammatory factors hs-CRP and IL-6, and it can also reduce the levels of CK-MB, cTnT, E-selection (ES), and H-FABP, and alleviate myocardial injury. Shenqi injection (68, 70) can reduce the myocardial enzymes lactate dehydrogenase (LDH), aspartate aminotransferase (AST), alanine aminotransferase (ALT), CK-MB, Hydroxybutyrate dehydrogenase (HBDH), and myocardial fibrosis TGF-β1, CTGF, Recombinant Procollagen I C-Terminal Propeptide (PICP), type I pro-hydroxy terminal cross linked peptide (CITP), and PIIINP related indicators, and reduce myocardial fibrosis to protect cardiomyocytes. Shenmai injection (66), Astragalus injection (65), and Xinmailong injection (54) can reduce cTnI levels. Astragalus injection (52, 65) can improve ventricular remodeling, reduce pulmonary artery systolic pressure (SPAP), increase PaO2, SaO2, force expiratory volume in 1 s (FEV1), and effectively expand blood vessels, improve pulmonary microcirculation and systemic hypoxia, and reduce heart load. Xinmailong injection (58) can improve pulmonary artery systolic pressure (PASP), mean pulmonary artery pressure (MPAP), endothelin (ET), nitric oxide (NO), thromboxane (TXA2), and prostacyclin (PGI2), reduce pulmonary artery pressure, and alleviate vascular endothelial function damage. Salviae Miltiorrhizae and Ligustrazine Hydrochloride injection (67) increased systolic blood pressure (SBP), diastolic blood pressure (DBP), and estimated glomerular filtration rate (eGFR) levels, decreased plasma renin activity, angiotensin II, serum creatinine (SCr), and aldosterone (ALD) levels, and inhibited the activation of renin-angiotensin-aldosterone system (RAAS).

#### 2.2.4 Other traditional alternative strategies for RHF treatment

One of the unique advantages of traditional Chinese medicine treatment lies in the combination of internal and external treatments. In addition to internal treatment, external treatment methods, such as acupuncture, Tai Chi exercise, Baduanjin exercise, and acupoint application, also play a role in the treatment of heart failure. A systematic review and meta-analysis of 32 RCTs involving 2,499 patients revealed that integrated acupuncture and Western medicine therapies were more effective than Western medicine treatments alone regarding the indicators of efficacy rate, LVEF, improved 6MWD, MLHFQ, and CO (71). Multiple meta-analyses showed that heart failure patients took Tai Chi rehabilitation exercise on the basis of conventional drug treatment. After 6 months of treatment, quality of life, cardiac function grading, 6MWD, LVEF, and LVEDD in the treatment group was significantly better than that of the control group (72, 73). Clinical research (74, 75) reported that, compared with taking conventional drugs alone, conventional Western medicine combined with Ba Duan Jin's comprehensive treatment can significantly improve patients’ BNP, metabolic equivalents (METs), maximum oxygen consumption (VO_2_Max), 6MWD, New York Heart Association (NYHA) classification, LVEF, TCM symptoms, quality of life, and other indicators, and the degree of improvement is obviously better than that of the control group. A clinical study by Huang et al. (76) reported that acupoint application can significantly improve clinical symptoms, cardiac function, and quality of life, and reduce the level of NT-proBNP.

In summary, the clinical evidence suggests that TCM is beneficial for the treatment of RHF. First, TCM improves the clinical symptoms of patients, including alleviating symptoms such as dyspnea, cough and expectoration, chest tightness and shortness of breath, shortness of breath, palpitation and palpitation, and intractable edema, while improving cardiopulmonary function. It also attenuates signs of global heart failure, such as jugular vein irritability and purple forceps, and can improve the patient's 6-min walking distance and exercise tolerance. It can improve the symptoms of traditional Chinese medicine, the total effective rate of clinical efficacy, and the quality of life of patients, shorten the length of stay and reduce the rate of readmission. Second, TCM improves cardiac function, including increased LVEF levels and FS levels, and decreased LVEDV, LVESD, and LVESV levels, increased SV, CO, and CI levels, and enhanced cardiac contractility. Third, TCM improves biochemical parameters as follows: lowers indexes of myocardial zymography, LDH, AST, ALT, CK-MB, HDBH, and decreases myocardial injury; decreases SPAP, increases PaO_2_, SaO_2_, and FEV1, improves microcirculation and systemic hypoxia in the lungs, and reduces cardiac workload; preserves endothelial function, reduces ET-1 levels, increases NO levels, and suppresses inflammatory responses; decreases the level of hs-CRP; improves lipids, including lower TG, TC, and LDL-C levels and higher HDL-C levels; inhibits ventricular remodeling and decreases TGF, a marker of myocardial fibrosis-β1. CTGF, PICP, CITP and PIIINP levels.

## 3 The mechanism of TCM in treating refractory heart failure

**Figure 2:**
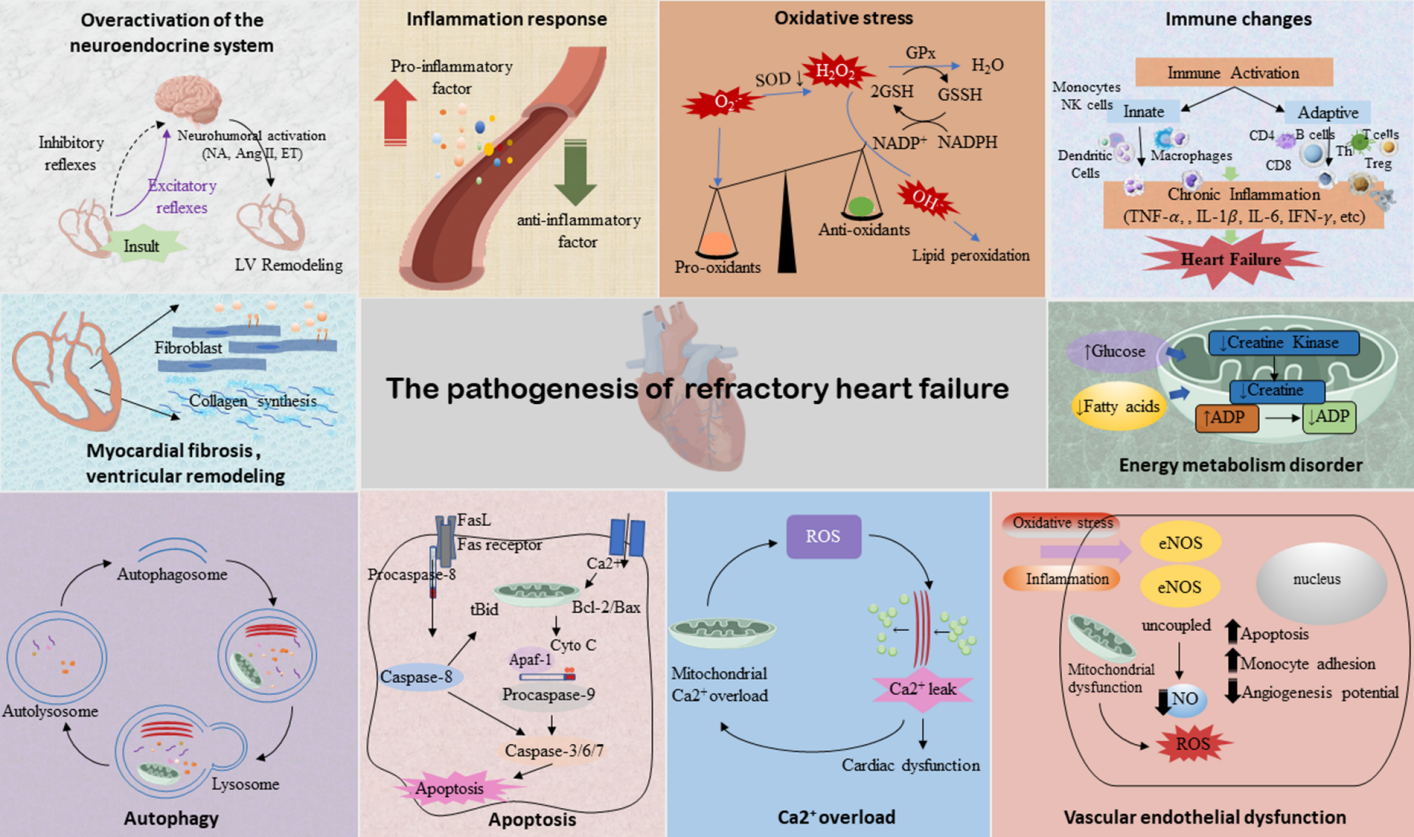
The pathogenesis of RHF. Illustrations by Figdraw.

**Figure 3:**
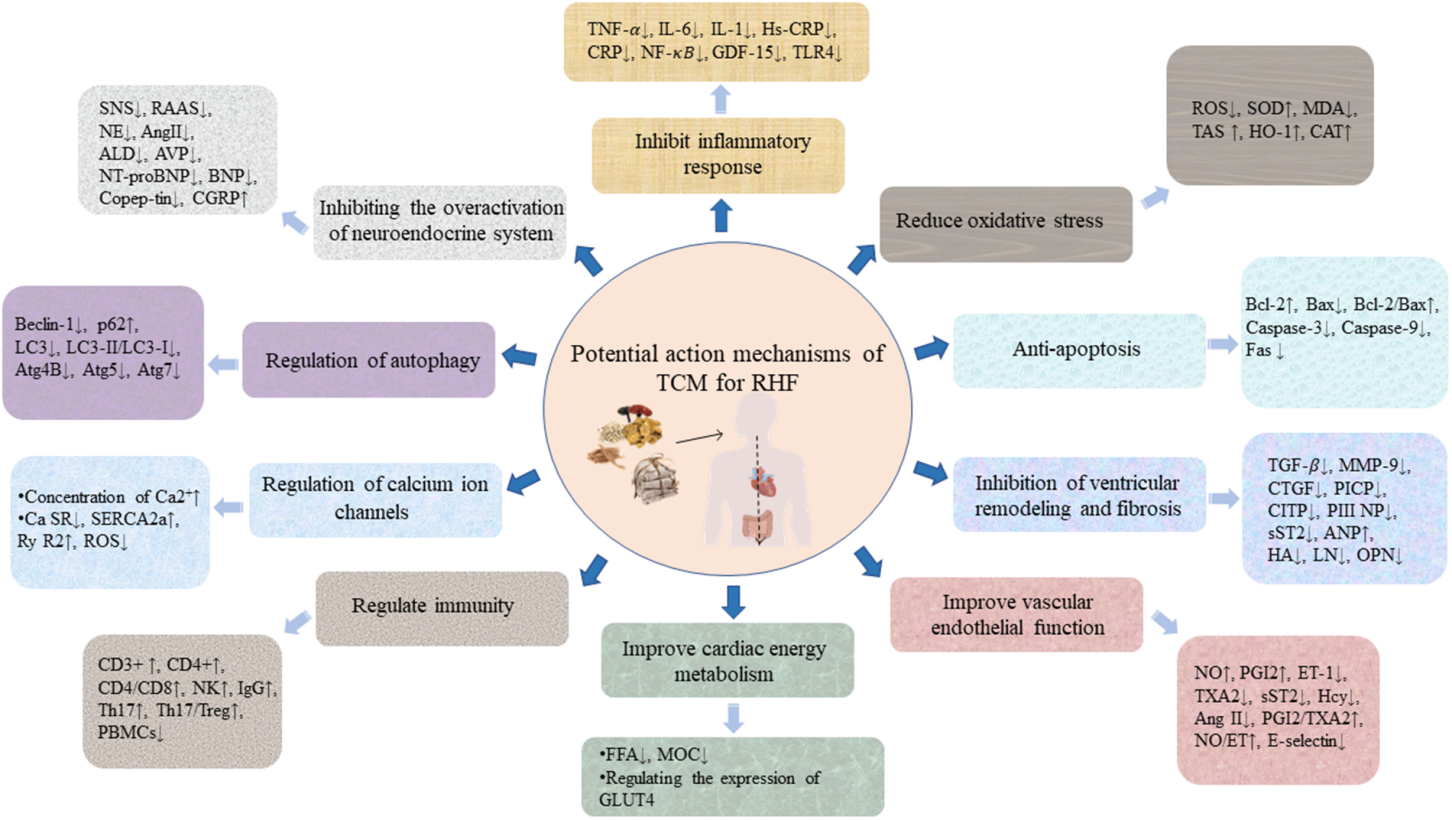
The potential action mechanisms of TCM for RHF. Illustrations by Figdraw.

The pathogenesis of RHF has not been fully elucidated. Current research shows that neuroendocrine mechanisms, mainly including enhanced excitability of the sympathetic nervous system (SNS), activation of RAAS, and ventricular remodeling play important roles in the progress of RHF. In addition, cardiomyocyte energy supply and utilization disorders, inflammatory mediators, oxidative stress, vascular endothelial cell dysfunction, intracellular Ca^2+^ overload, and cardiomyocyte apoptosis are also involved in the pathophysiology of RHF (Figure 2), which are the key factors that make it deteriorate and cascade (77). The potential action mechanisms of TCM for RHF are shown in Figure 3.

### 3.1 Suppression of neuroendocrine system hyperactivation

During the pathogenesis of HF, the activation of the neuroendocrine system leads to myocardial remodeling, which is a key factor in the occurrence and development of HF. In the early stage of HF, the SNS and RAAS are activated, playing a certain compensatory role. With the excessive activation of the neuroendocrine system, negative systemic effects will be generated on various organs, causing hemodynamic stress, circulatory disorder, and ventricular remodeling, thereby accelerating the progress of HF (78, 79). At the same time, with the aggravation of HF and the long-term activation of the neuroendocrine system, the myocardial afterload and oxygen consumption will be increased, promoting myocardial hypertrophy and then further worsening of HF, and producing a series of adverse cardiovascular events in the subsequent development of the diseases (80, 81).

By inhibiting the activity of the RAAS, aconite downregulates the level of nerve-cytokines, significantly improves the neuroendocrine disorder in HF rats caused by abdominal aortic coarctation, relieves ventricular remodeling, and improves the symptoms of HF (82). Dong et al. (83), through an experimental study of the chronic heart failure (CHF) rat model established by narrowing the abdominal aorta, confirm that Zhenwu decoction can inhibit SNS by reducing the plasma norepinephrine (NE) level of rats in the administered group, and then effectively improve the cardiac function of rats with HF. Liu et al. (84) also confirm that Zhenwu decoction can significantly reduce the levels of serum Ang II and ALD in rats with HF, and fight cardiac failure by antagonizing the excessive activation of RAAS. Xinmailong injection can correct hemodynamic disorder in various ways, and has the effects of strengthening the heart, diuresis, pulmonary vascular dilation, reducing pulmonary artery pressure, improving microcirculation and ventricular remodeling, regulating humoral factors and neuroendocrine, and scavenging oxygen free radicals, etc., to correct the neuroendocrine imbalance and antagonize a ventricular remodeling effect (85). The Qiliqiangxin capsule can reduce the levels of serum NE and ALD, and inhibit the activation of SNS and RAAS, thus delaying ventricular remodeling and disease progression in patients with CHF (85).

### 3.2 Inhibition of inflammatory response

Normal cardiac function is maintained by a balanced homeostasis of pro-inflammatory and anti-inflammatory cytokines. Dysregulation of the inflammatory response induces cardiomyocyte hypertrophy, apoptosis, fibrosis, and changes in cardiac hemodynamics (86), ultimately accelerating the progression of HF. Several studies have proved that the increase of pro-inflammatory biomarkers in patients with HF is associated with disease severity (87), and reducing the expression of these inflammatory factors can effectively improve HF (86, 88–90). Therefore, inhibiting the release of inflammatory factors is of great significance for delaying ventricular remodeling and improving cardiac function.

Inflammatory cytokines related to RHF mainly include TNF-α, interleukin, nuclear factor κB (NF-κB), and other inflammatory cytokines, which play an important role in cardiac dysfunction and adverse cardiac remodeling. Besides inflammation, inflammatory mediators can also activate endothelial cytokines (91), form a driving network with the RAAS system, and jointly participate in the development of HF. The activation of NF-κB increases the production of inflammatory cytokines and chemokines. Zhang et al. (92) found that astragaloside can reduce the levels of inflammatory factors, such as IL-6, IL-1, and TNF-α, by regulating the TLR4/NF-κB/PPAR α signaling pathway, thus improving cardiac function and cardiomyocyte viability, and alleviating myocardial injury in HF with midrange EF (HFmrEF) mouse model induced by LPS. Experimental studies (93) have shown that the compatibility of aconite and licorice can relieve inflammation and ventricular remodeling in mice through the TLR4/NF-κB pathway. Qichenyiqi dropping pills (94) can reduce the expression of inflammatory cytokines TNF-α and IL-6, thereby inhibiting the function of inflammatory factor pathways TNF-α, NF-κB, and IL-6-STAT3, improving the hemodynamic of the HF model, downregulating the level of inflammatory cell pathways NF-κB and PNFKB1, and alleviating ventricular remodeling (95).

### 3.3 Reduction of oxidative stress

Oxidative stress refers to the process in which highly reactive molecules such as reactive oxygen species (ROS) and reactive nitrogen species (RNS) accumulate excessively and then exceed the oxide scavenging capacity, resulting in the imbalance between pro-oxidation and the antioxidant system in the body (96). It is an important inducing factor of myocardial injury. HF can lead to the excessive accumulation of ROS and lipid peroxide, which aggravates cell membrane damage and leads to myocardial contraction and diastolic function deterioration (97). In addition, ROS cannot activate a variety of signaling kinases and transcription factors to mediate apoptosis but can stimulate the proliferation of cardiac fibroblasts, and activate matrix metalloproteinase, causing an increase of the extracellular matrix to induce myocardial remodeling, which is an important pathogenesis of HF.

Many studies have proved that TCM can reduce myocardial damage through antioxidation. Chen et al. (98) established a CHF model of cardiac hypertrophy induced by AAS and found that Qishenyiqi dropping pills (QSYQ) can significantly inhibit the progression of cardiac hypertrophy to HF induced by AAS in rats; its mechanism is related to the improvement of energy metabolism, oxidative stress, apoptosis, and other pathways. Lingguizhugan decoction can affect the SIRT1-AMPK-PGC-1α pathway, restore mitochondrial membrane potential, reduce ROS and malondialdehyde (MDA) levels, alleviate mitochondria and oxidative stress damage, promote mitochondrial biogenesis, increase the ejection fraction, and improve cardiac function in rats with HF (99). At the same time, it also antagonizes oxidative stress damage through the Nrf2/Keap1/HO-1 pathway (100). In addition, after different doses of Qiliqiangxin capsule intervention in rat models of chronic heart failure, the LDH and ROS levels in the serum of rats with HF are decreased, the activity of superoxide dismutase (SOD) is enhanced, the expression of the pro-apoptotic protein is inhibited, and the expression of B-cell lymphoma-2 (Bcl-2), p-Akt/Akt and p-GSK3β/GSK3β is upregulated. The cell experiments also demonstrate that, after hydrogen peroxide (H_2_O_2_) damage to cardiomyocyte (H9C2), cell proliferation is significantly improved, SOD activity, heme oxygenase-1 (HO-1), and catalase (CAT) mRNA expressions are significantly upregulated, while LDH, ROS, and apoptosis are significantly inhibited after 24 h under the intervention of Qiliqiangxin ultrafine powder. At the same time, it also inhibits mitochondria division, the opening of the mitochondrial membrane permeability transition pore (mPTP) and the decline of MMP, and alleviates the mitochondria-dependent apoptosis of cardiomyocytes damaged by oxidative stress (101). Ginsenoside Rb 1 can improve the hypoxia tolerance of cardiomyocytes by mediating the AMPK signaling pathway, reducing the levels of Atg4B, Atg5, Beclin1, Atg 7, LC3BII, and LC3BII/I ratio, and inhibit excessive autophagy to protect cardiomyocytes from hypoxic injury (102).

### 3.4 Inhibition of cardiomyocyte apoptosis and pyroptosis

Apoptosis can promote the progression of various cardiovascular diseases and is one of the important pathological mechanisms of myocardial tissue injury. In the failing heart, the decrease in the number of ventricular cardiomyocytes and the apoptosis of cardiomyocytes caused by interstitial fibrosis not only participate in ventricular remodeling, but is also one of the important reasons closely related to the severity of HF (103). Pyroptosis is a type of programmed cell death accompanied by inflammation, whose features are the formation of membrane pores on the cytoplasmic membrane, release of pro-inflammatory factors and cell contents, and swelling and rapid rupture of cells (104). Recent studies have suggested that pyroptosis is closely related to CHF; therefore, targeting pyroptosis has a good prospect in alleviating CHF (105). Therefore, inhibiting cardiomyocyte apoptosis and pyroptosis, intervening with the cause of apoptosis, and blocking the pathway of inducing cardiomyocyte apoptosis are of great significance in preventing the occurrence of HF and the deterioration of cardiac function (106).

Basic research has shown that Shenfu Qiangxin pills can significantly reduce myocardial hypertrophy in rats with chronic binding stress, inhibit ventricular septal thickness, and left ventricular posterior wall hypertrophy, reduce the expression of mRNA of damaged tissue pattern receptors, inhibit the expression of extracellular regulatory protein kinases 1/2 and p38 protein of mitogen-activated protein kinase signaling pathway, upregulate Bcl-2 expression, inhibit associated X protein (Bax) expression, and reduce the rate of cardiomyocyte apoptosis, exerting a protective role on the heart (107, 108). By downregulating the expression of the pro-apoptotic protein Bax, upregulating the anti-apoptotic protein Bcl-2 expression and the Bcl-2/Bax ratio, Yiqi Huoxue formula can improve the cardiac function of rats with myocardial infarction, thereby alleviating the myocardial apoptotic injury and protecting the heart (109). The Qiliqiangxin capsule activates the PI3K/AKT/Gsk3β signaling pathway by decreasing ROS and downregulating the expression of apoptosis-related proteins Fas and caspase-3, and inhibits the apoptosis of cardiomyocytes of rats with chronic heart failure (101). Shengmai injection can alleviate endoplasmic reticulum stress, upregulate Bcl-2/Bax ratio, downregulate caspase-3 expression, inhibit caspase-dependent apoptosis, and then protect the myocardial damage caused by doxorubicin (110, 111). Fan et al. (112) report that Shenfu injection can improve CHF by regulating pyroptosis based on the NLRP3/caspase-1 pathway. There are also studies that Shenkui Tongmai Granule can improve the cardiac function of the CHF rat model and reduce the pyroptosis of cardiomyocytes (113). Studies have shown that excessive or continuous endoplasmic reticulum stress promotes the occurrence and development of chronic heart failure by inducing cardiomyocyte apoptosis (114). Luo et al. (115) report Yiqi Huoxue Recipe improves heart function through inhibiting apoptosis related to endoplasmic reticulum stress in a myocardial infarction model of rats.

### 3.5 Inhibition of ventricular remodeling and fibrosis

The alteration of ventricular structure and myocardial fibrosis are the key factors causing the further progression of RHF. It has been found that the deposition of a large amount of extracellular matrix and collagen changes the ventricular structure along with the increase of the chamber volume, damages the myocardial structure, causes cardiac diastolic and systolic dysfunction, reduces cardiac function, and promotes the development of HF (116). Therefore, in addition to the improvement of existing clinical symptoms, patients with RHF should focus on delaying the process of ventricular remodeling and myocardial fibrosis.

A number of basic and clinical studies have shown that TCM can effectively delay cardiac remodeling and improve myocardial fibrosis. Zhen et al. (117) discovered that the Qiliqiangxin capsule can effectively improve the clinical symptoms of patients with HF, reduce the serum levels of MMP-2, MMP-9, and MMP-13, increase the levels of tissue inhibitor of matrix metalloproteinases (TIMP)-1 and TIMP-2, regulate the expression of MMPs and TIMPs in patients with HF, and improve the metabolic disorders of the extracellular matrix, thereby inhibiting myocardial fibrosis and ventricular remodeling and improving cardiac function. Another study has reported that astragaloside can improve myocardial ultrastructural damage, reduce myocardial collagen deposition in rats with HF (118), improve myocardial fibrosis, improve cardiac function, and inhibit the development of CHF (119). Li et al. (120) used Ang II to induce the CHF model when studying the intervention effect of Shengmai injection on animals with chronic heart failure and discovered that Shengmai injection can reduce cardiomyocyte hypertrophy and apoptosis, protect mitochondrial function in hypertrophic cardiomyocytes, and inhibit cardiac fibrosis. Wei et al. (121) applied the Isoproterenol(ISO)-induced myocardial injury model to study the effect mechanism of compound Danshen dropping pills and found that it can significantly improve the survival rate of ISO-treated rats and alleviate myocardial fibrosis, whose mechanism is associated with improving the metabolism of myocardial tissue. The Qiliqiangxin capsule alleviates cardiac remodeling by inhibiting the TGF-β1/Smad3 and NF-κB signaling pathways (122). Shenfuyixin granules can downregulate the expression of c-fos and c-myc and delay myocardial remodeling in HF rats (123).

### 3.6 Improvement of vascular endothelial function

Endothelial dysfunction is the pathophysiological basis of HF (124). Cardiac endothelial cells not only participate in the formation and metabolism of blood vessels but also regulate myocardial fibrosis, cardiac hypertrophy, and cardiomyocyte apoptosis and autophagy. When HF occurs, endothelial cell dysfunction not only causes a decrease in NO and an increase in ET, leading to increased vascular resistance, but also the damaged endothelial cells will produce a large number of growth factors and active substances and promote the proliferation of smooth muscle cells and the production of the matrix (125), resulting in systemic peripheral resistance to decrease vascular compliance, vascular stiffness, and arterial dilation damage, aggravate vascular remodeling, cause heart inflammation and myocardial fibrosis, and promote the occurrence and development of HF (126).

TCM can reduce arterial wall stiffness and vascular resistance, and slow down ventricular remodeling by protecting vascular endothelial function. Studies have found that the Shensongyangxin capsule can not only reduce the damage of oxidative stress and immune inflammation on vascular endothelial function by inhibiting the formation of oxygen free radicals and the body's inflammation, but also inhibit the secretion of angiotensin and catecholamine, promote the release of vasodilator factor, regulate the body's vasomotor balance, and protect vascular endothelial function (127). Zhang and Huang (128) studied the effect of the Qiliqiangxin capsule on myocardial injury and vascular endothelial function in patients after percutaneous coronary intervention, and found that the Qiliqiangxin capsule may protect the microvascular endothelial function, reduce myocardial injury, and improve cardiac function by increasing plasma NO and reducing ET-1. In addition, in animal experiments, the Qiliqiangxin capsule can improve the serum concentrations of VEGF and HIF-1 in HF rats, and activate the angiogenesis pathway associated with HIF-1-VEGF, thereby promoting angiogenesis, alleviating myocardial ischemia and hypoxia injury, and delaying ventricular remodeling. The Guanxinkang capsule combined with ivabradine hydrochloride tablets can regulate the levels of serum Ang II, ACE II, and NT-pro BNP, reduce vascular endothelial cell damage in patients with RHC, and improve cardiac function (49). The Shenfu injection enhances eNOS activity through the PI3K/Akt signaling pathway, to promote vasodilation and improve the microcirculation in the failing heart (129).

### 3.7 Improvement of cardiac energy metabolism

The heart is the most active organ with high oxygen consumption and high energy consumption in the human body. The process by which cardiomyocytes can use a variety of energy substrates to store and utilize energy is called myocardial energy metabolism (130). Normal myocardial energy metabolism can maintain a stable environment in cardiomyocytes and keep a normal diastolic function. Studies have found that patients with HF will exhibit serious energy metabolism disorders, including substrate absorption and utilization disorders, mitochondrial structure and function abnormalities, and myocardial high-energy phosphate changes, resulting in insufficient heart energy supply, which will lead to heart pump dysfunction and systemic energy metabolism disorders, thus causing the change of heart structure and function and speeding up the development of HF (131). Numerous studies have shown that there is abnormal energy metabolism in HF, and the regulation of myocardial energy metabolism may be a new treatment for HF (132).

Experimental studies have proved that the Shenfuqiangxin mixture can upregulate the expression of phosphorylated AMPK (p-AMPK) and PGC-1α in rats with HF, reduce the levels of serum BNP, free fatty acid (FFA) and LDH in HF rats, regulate glucose transport and fatty acid oxidation metabolism, and accelerate myocardial energy metabolism (133). Shenfu formula, through activating AMPK-mediated fatty acid and glucose metabolism in the heart, regulates the gene expression of glucose transporter 4 (GLUT4) and promotes the myocardial uptake and utilization of cardiac glucose, thus improving the myocardial energy metabolism to alleviate heart failure (134). Shenmai injection maintains the mitochondrial structure and function, improves energy metabolism, and increases the survival rate of cardiomyocytes by maintaining mitochondrial membrane potential and inhibiting the opening of mitochondrial permeability transition pore (135). Han et al. (136) use the AAC model to study its mechanism and find that the potential mechanism of Yiqifumai in treating HF is realized by enhancing myocardial mitochondrial function and regulating myocardial energy metabolism.

### 3.8 Regulation of immunity

Studies have found that many types of immune cells, such as NK cells, T cells, B cells, monocytes, macrophages, etc., are all involved in the process of cardiac fibrosis and myocardial remodeling (137). Therefore, the activation of immune cell-mediated immunity may play a crucial role in the pathogenesis of various forms of RHF. The treatment targeting immune regulation is considered beneficial in patients with HF with immune disorders that are a key factor in the aggravation of RHF (138).

A study found that the Shenfu injection can reduce the levels of plasma IL-10 and NT-pro BNP in patients with HF, and increase the proportion of TGF-β1, peripheral blood CD4+, and CD4+CD+25Foxp3+Treg cells, thereby exerting an immune regulatory role and then delaying the development of HF (139). The Qiliqiangxin capsule can significantly increase the levels of CD4+cell subsets, NK cells and IgG in patients with HF, improve ventricular remodeling and related immune indexes, and improve immunity and patients’ quality of life (140). The Lingguizhugan decoction can reduce the serum levels of IL-6, IL-18, and TNF-α in patients with HF, increase the levels of CD3+T, CD4+T, and CD4+T/CD8+T, and improve the cardiac function and clinical symptoms of patients by regulating immunity and inhibiting inflammatory response (141). The Qishanyiqi dropping pills can affect the dynamic balance of immune cell subsets and increase the levels of CD4 and CD4/CD8, thereby improving the immune function of patients with HF (142). The Shenqi compound can effectively regulate the disorder of CD4+T lymphocyte subsets in the peripheral blood of patients with HF, increase the levels of Th17, Th17/Treg and the serum IL-10, and reduce the expression of IL-2, IL-6, IL-17, TNF-α, and IFN-γ pro-inflammatory factors. At the same time, in animal experiments, the Shenqi compound can also reduce inflammatory damage, improve cardiac function in rats, and alleviate ventricular remodeling by inhibiting the IL-6/STAT3 signaling pathway and the differentiation of Th17 cells, and restoring the balance of the Th17/Treg cell (143).

### 3.9 Regulation of calcium ion channels

As the “second messenger” of cells, calcium ions (Ca^2+^) play an important role in the pathogenesis of cardiovascular diseases, and the imbalance of Ca^2+^ homeostasis in cardiomyocytes is a key factor in the pathological changes of HF. We all know that the essence of HF is the contraction or diastole dysfunction of cardiomyocytes, and the contraction and diastole of the heart are mainly carried out through the exchange of Ca^2+^ inside and outside the cell. When calcium homeostasis occurs in patients with HF, the intracellular Ca^2+^ homeostatic order will cause contraction and diastolic dysfunction by interfering with RyR2, the sarcoplasmic Ca^2+^-ATP enzyme (SERCA) pathway, and the sodium/calcium exchange protein (NCX) (144, 145), resulting in the development of HF.

TCM can repair abnormal calcium homeostasis in failing cardiomyocytes by regulating the expression and activity of various calcium-treated proteins. For example, ginsenoside Rg1 can regulate the intracellular Ca^2+^ concentration, reduce the expression of Ca SR, and play a myocardial protective role through the Ca^2+^/Ca SR pathway (146). Astragaloside can inhibit Ca^2+^ accumulation induced by LPS, prevent LPS-induced cardiac hypertrophy by inhibiting the Ca^2+^/CaN/NFAT-3/GATA-4 signaling pathway, and increase myocardial contractility (147). Liggustrazine can not only scavenge oxygen free radicals, but also inhibit lipid peroxidation and inflammation, maintain the dynamic balance of Ca^2+^, inhibit cell death, and protect cardiomyocytes (148). Qishen granule treatment can effectively reduce the concentration of Ca^2+^ in the cardiomyocytes of rats with HF, and improve cardiac function, ventricular remodeling, and lipid metabolism (149). Wenyang Yiqi Huoxue decoction can improve cardiac dysfunction in rats with HF by enhancing the activity of the Na^+^-K^+^-ATP and Ca^2+^-ATP enzymes and upregulating their mRNA and protein expressions (150).

### 3.10 Regulation of autophagy

Autophagy is a highly conserved dynamic change process in cell life that maintains cellular energy balance and organelle renewal (151), and is an important mechanism for maintaining the stability and function of the environment in cardiomyocytes, which can limit cardiac injury under many pathological conditions. However, increased autophagic flux in cardiomyocytes may cause autophagic cell death, leading to uncontrolled degradation of materials or an imbalance with lysosomal degradation, which is accompanied by excessive accumulation of autophagosomes, eventually leading to cell death (152). Several studies have found that autophagy in cardiomyocytes with high activation for a long period of time is one of the key factors leading to the progression of HF (153, 154). Therefore, regulating and stabilizing moderate autophagy in cardiomyocytes is important for the maintenance of normal cardiac function.

In animal experiments, Liao et al. (155) found that the Shenfu injection can activate the PI3K/Akt/mTOR signaling pathway, downregulate the content of autophagosome marker microtubule-related protein 1 light chain 3 (LC3) of myocardial tissue, increase the expression level of autophagy marker protein p62 to inhibit autophagy, and reduce myocardial fibrosis, thereby protecting the damaged cardiomyocytes and improving heart function. The PINK1/Parkin pathway is a typical mitochondrial autophagy pathway and an important regulator in the process of mitochondrial autophagy (156). Many studies have found that the activation of the PINK1/Parkin pathway can prevent mitochondrial damage and cardiomyocyte apoptosis by increasing the level of mitochondrial autophagy (157), improve cardiac contractile function (158), and reduce heart failure(159). There are studies (160, 161) reporting that Nuanxinkang can prevent the development of myocardial infarction-induced chronic heart failure by promoting PINK1/Parkin-mediated mitophagy and ginsenoside Rg1 can protect against cardiac remodeling in heart failure via SIRT1/PINK1/Parkin-mediated mitophagy.

In summary, the mechanism of TCM in treating RHF includes, but is not limited to, the following: (1) inhibiting the overactivation of the neuroendocrine system by targeting to reduce the levels of serum NE, AngII, ALD, etc.; (2) regulating the immune and inflammatory responses by targeting inflammatory cytokines, such as TNF-α, IL-6, IL-1β, IL-2β, NF*-*κB, CD3+ T, CD4+ T, etc.; (3) enhancing myocardial mitochondrial function, maintaining a Ca^2+^ dynamic balance, improving the myocardial energy metabolism and inhibiting myocardial damage by balancing myocardial energy metabolism, including reducing the content of lactic acid and free fatty acids, promoting the transport and utilization of fatty acids, and mediating the metabolism of fatty acids and glucose in the heart; (4) improving extracellular matrix metabolism disorders, and inhibiting myocardial fibrosis and ventricular remodeling by targeting the expression of MMPs and TIMPs, and upregulating the expression of LCAD, LVMI, CVF, LVPWD, PFK1 protein, and mRNA; (5) alleviating myocardial damage caused by hypoxia-induced apoptosis and oxidative stress through targeted regulation of LDH, ROS, SOD, HO-1, CAT, Fas, and caspase-3 expression, as well as the Bcl-2/Bax ratio; (6) activating the HIF-1-VEGF angiogenesis pathway and alleviating the endothelial cell dysfunction by targeting NO, PGI2, and ET-1; and (7) regulating mitochondria and cell autophagy activity to play a role in myocardial protection by targeting key autophagy proteins such as Atg4B, Atg5, Atg7, Beclin1, mTOR, LC3-II, LC3-I, and p62.

## 4 Conclusion and perspectives

RHF is a serious clinical problem that imposes a significant economic burden on a patient's family and society. Due to the increasing aging population, the prevalence of RHF has climbed annually and the difficulty finding treatment has also increased, which has gradually led to a long treatment cycle, persistent symptoms and signs, a poor prognosis, and other clinical characteristics. Currently, there are no large randomized clinical trials with RHF as the research subject and cardiovascular events as the observation endpoint; therefore, the treatment modalities that can reduce the cardiovascular event rate of RHF are unclear, and modern medicine still lacks effective methods of treating RHF. As a result, how to take effective measures to improve the survival rate of patients with RHF is a current medical dilemma that urgently needs to be addressed.

Guideline-directed medical therapy (GDMT) is the fundamental treatment for RHF. In recent years, clinical trials for sacubitril valsartan, ivabradine, levosimendan, and sodium glucose cotransporter 2 inhibitors have been released, which can benefit patients in reducing the risk of death and readmission rates (162–165). The standardized application of GDMT has improved the overall prevention and control of RHF. After the appropriate application of various treatment measures based on GDMT, if the patient's heart function is still severely damaged and there are no other effective treatment methods, heart transplantation is the most effective treatment. However, the shortage of heart donors and the ischemic time of donors restrict the possibility of heart transplantation (166). In addition, complications, such as thrombosis, bleeding, infection, and those caused by mechanical devices used in heart transplantation, are still difficult to avoid, and it is still necessary to continue to explore the fully built-in device and appropriate anti-thrombotic scheme in the future. In China's long-term medical practice, traditional Chinese medicine has been widely used in the treatment of heart failure due to its unique efficacy and safety advantages, accumulating rich experience. The combination of traditional Chinese and Western medicine in the treatment of heart failure is currently a research hotspot. With the emergence of research results, the efficacy of traditional Chinese medicine in treating heart failure has gradually been confirmed. Reports have shown that the combined treatment of syndrome differentiation based on a Chinese herbal decoction, CPMs, and Chinese medicine injection, which is based on the conventional treatment of Western medicine, can effectively alleviate the clinical symptoms, improve the quality of life and exercise tolerance, improve the long-term prognosis, and reduce the rate of rehospitalization and mortality in patients with RHF.

TCM has provided new ideas and directions for the treatment of RHF; however, several problems have also been identified. First, it is difficult to apply TCM widely clinically: TCM shows certain clinical advantages through syndrome differentiation and individualized treatment of RHF, and because of the flexibility of syndrome differentiation, there is no uniform protocol for syndrome differentiation and treatment, making it difficult to apply TCM widely in the clinic. Second, there are few high-quality clinical studies: on the one hand, the clinical sample size is small, the observation period is short, and large-scale, multicenter, prospective randomized controlled studies are lacking; on the other hand, the clinical protocol design was not rigorous, and some of the included clinical studies had unclear descriptions of sample size estimation, random allocation concealment scheme, blinded design, and dropout rate, resulting in generally low-quality articles. Third, clinical efficacy is unclear: although clinical studies have focused on RHF, the results of large sample, multicenter, prospective RCTs focusing on RHF with cardiovascular events as the outcome measure are still lacking, and the treatment of RHF is still limited to improving clinical symptoms. Therefore, it is unclear which treatment modality can reduce the cardiovascular event rate of RHF. Finally, there is an unclear mechanism of action: TCM has a complex composition, and its mechanism of action in treating RHF is unclear. In addition, animal models of RHF that meet the clinical characteristics are currently lacking. In response to the above scientific questions, we suggest future research should focus on the following directions: (1) strengthening the scientific and normative of clinical research, carrying out multicenter and large sample high-quality RCTs with cardiovascular events as the observed endpoints, establishing treatments with evidence-based medical evidence, and provide more reliable clinical evidence for the Chinese medicine treatment of RHF; (2) establishing an animal model of RHF that conforms to clinical characteristics; (3) combining the use of network pharmacology, bioinformatics, transcriptomics, proteomics, metabolomics, UPLC-Q/Orbitrap/MS, and other techniques to deeply mine the deep action mechanisms of TCM for the treatment of RHF to provide a scientific basis for its clinical application.

In conclusion, RHF remains an urgent clinical problem. TCM has great potential for treating RHF, but further systematic and in-depth clinical and basic experimental studies are still needed.

## References

[1] Cappannoli L Scacciavillani R Rocco E Perna F Narducci ML Vaccarella M Cardiac contractility modulation for patient with refractory heart failure: an updated evidence-based review. Heart Fail Rev. (2021) 26(2):227–35. 10.1007/s10741-020-10030-4 32974722

[2] Fang JC Ewald GA Allen LA Butler J Westlake Canary CA Colvin-Adams M Advanced (stage D) heart failure: a statement from the Heart Failure Society of America Guidelines Committee. J Card Fail. (2015) 21(6):519–34. 10.1016/j.cardfail.2015.04.013 25953697

[3] GBD 2017 Disease and Injury Incidence and Prevalence Collaborators. Global, regional, and national incidence, prevalence, and years lived with disability for 354 diseases and injuries for 195 countries and territories, 1990–2017: a systematic analysis for the global burden of disease study 2017. Lancet. (2018) 392(10159):1789–858. 10.1016/S0140-6736(18)32279-7 30496104 PMC6227754

[4] Yancy CW Jessup M Bozkurt B Butler J Casey DE Jr Drazner MH 2013 ACCF/AHA guideline for the management of heart failure: a report of the American College of Cardiology Foundation/American Heart Association Task Force on Practice Guidelines. J Am Coll Cardiol. (2013) 62(16):e147–239. 10.1016/j.jacc.2013.05.019 23747642

[5] Yancy CW Jessup M Bozkurt B Butler J Casey DE Jr Colvin MM 2016 ACC/AHA/HFSA focused update on new pharmacological therapy for heart failure: an update of the 2013 ACCF/AHA guideline for the management of heart failure: a report of the American College of Cardiology/American Heart Association Task Force on Clinical Practice Guidelines and the Heart Failure Society of America. J Am Coll Cardiol. (2016) 68(13):1476–88. 10.1016/j.jacc.2016.05.011 27216111

[6] Abouezzeddine OF Redfield MM. Who has advanced heart failure? Definition and epidemiology. Congest Heart Fail. (2011) 17(4):160–8. 10.1111/j.1751-7133.2011.00246.x 21790965 PMC3857759

[7] Vincenzi A Cesana F Ciro A Garatti L Achilli F. Sacubitril/valsartan in “field practice” patients with advanced heart failure: a monocentric Italian experience. Cardiology. (2017) 138(Suppl 1):13–6. 10.1159/000484877 29262402

[8] Sabouret P Galati G Angoulvant D Germanova O Castelletti S Pathak A The interplay between cardiology and diabetology: a renewed collaboration to optimize cardiovascular prevention and heart failure management. Eur Heart J Cardiovasc Pharmacother. (2020) 6(6):394–404. 10.1093/ehjcvp/pvaa051 32402065

[9] Zhang MD Bi YF Wang XL Wang S Zhang X Liu Y Literature analysis on the characteristics of Chinese medicine syndromes of refractory heart failure. Asia Pac Trad Med. (2023) 19(01):165–8. 10.11954/ytctyy.202301038

[10] Jiao Q Li ZN Chen HC Yang JM Ma J Sun D Treatment of refractory heart failure based on Fuyang. Chin J Integr Med Cardio Cerebrovasc Dis. (2023) 21(09):1714–6. 10.12102/j.issn.1672-1349.2023.09.036

[11] Li H Li B Li WX. The effect of Shenlu Ningxin decoction on refractory heart failure and its effect on myocardial remodeling and cardiac function in patients with refractory heart failure. Glob Trad Chin Med. (2022) 15(02):338–41. 10.3969/j.issn.1674-1749.2022.02.037

[12] Dong ML. Clinical effect of modified Linggui Zhugan decoction on intractable heart failure. Chin J Clin Ration Drug Use. (2022) 15(14):4–7. 10.15887/j.cnki.13-1389/r.2022.14.002

[13] Zhang RZ. Clinical effect of Shenqi Qiangxin decoction and Wenyang Lishui decoction on refractory heart failure. New Chin Med. (2021) 53(06):38–41. 10.13457/j.cnki.jncm.2021.06.009

[14] Chen AH. Clinical observation of Danlou Zhenwu decoction in the treatment of refractory heart failure. Chin J Integr Med Cardio Cerebrovasc Dis. (2020) 18(02):281–3. 10.12102/j.issn.1672-1349.2020.02.021

[15] Tian ZL. Clinical effect observation of modified Wenyang Huayu Lishui decoction in the treatment of refractory heart failure. Inner Mong J Trad Chin Med. (2019) 38(11):13–4. 10.16040/j.cnki.cn15-1101.2019.11.007

[16] Zhao D Li C Li J. Clinical study on Zhenwu Zhuyu decoction in the treatment of refractory heart failure. Chin J Integr Med Cardio Cerebrovasc Dis. (2019) 17(08):1236–8. 10.12102/j.issn.1672-1349.2019.08.034

[17] Zhu J Liu YY. Clinical observation of Yiqi Qiangxin Lishui formula in the treatment of refractory heart failure. Xinjiang J Trad Chin Med. (2016) 34(04):27–8.

[18] Yang L Liu YG Ding XM Wang XM. Clinical observation on 30 cases of refractory heart failure treated with Jiawei Yixin decoction. Hunan J Trad Chin Med. (2017) 33(03):42–4. 10.16808/j.cnki.issn1003-7705.2017.03.018

[19] Wu H. Warming Yang for diuresis prescription combined with western medicine in the treatment of severe refractory heart failure for 30 cases. Chin Med Modern Distance Educ China. (2016) 14(22):95–6. 10.3969/j.issn.1672-2779.2016.22.042

[20] Cao RF. Clinical observation on the treatment of refractory heart failure with Yang deficiency and water generosis with self-prepared Xinshuai formula. Chin Foreign Med Res. (2017) 15(36):169–71. 10.14033/j.cnki.cfmr.2017.36.087

[21] Wei YM. Treating 50 cases of refractory heart failure with the Yiqi Qiangxin prescription plus western medicine. Clin J Chin Med. (2020) 12(10):74–6. 10.3969/j.issn.1674-7860.2020.10.026

[22] Huo JY. Observation on the curative effect of Yiqi Qiangxin decoction in the treatment of refractory heart failure. Shaanxi J Trad Chin Med. (2016) 37(08):985–6. 10.3969/j.issn.1000-7369.2016.08.021

[23] Qin Y Li F Liu YQ. Effect observation of Zhenwu Zhuyu decoction in the treatment of refractory heart failure. Guizhou Med J. (2020) 44(10):1590–1.

[24] Xu YF Song SZ. Clinical observation of Poge Jiuxin decoction combined with western medicine in treatment of refractory heart failure. World Latest Med Inform. (2018) 18(44):3–4. 10.19613/j.cnki.1671-3141.2018.44.002

[25] Zhang HL Zhang T. Clinical efficacy of integrated traditional Chinese and western medicine in the treatment of refractory heart failure. Curr Med Res Pract. (2018) 3(16):139–40. 10.19347/j.cnki.2096-1413.201816066

[26] Ji WS. Clinical study on Shenqi Jidan Lizhi long decoction in treatment of refractory congestive heart failure patients. Med J Chin People’s Health. (2016) 28(06):93–4. 10.3969/j.issn.1672-0369.2016.06.046

[27] Lu YJ Li J Zhao W. Effect of Guipi decoction combined with milrinone on LVEF and NT-proBNP levels in patients with refractory heart failure. Health Med Res Pract. (2019) 16(06):61–3. 10.11986/j.issn.1673-873X.2019.06.015

[28] Wu GJ Yao ZQ Zhang XW Li GF. Effect of modified Fangji Fuling decoction on PBMCs orphan nuclear receptor and ventricular remodeling in patients with refractory heart failure. Clin J Chin Med. (2022) 14(30):43–5. 10.3969/j.issn.1674-7860.2022.30.014

[29] Wang YR. Clinical observation on treatment of refractory heart failure with integrated traditional Chinese and western medicine. J Pract Trad Chin Med. (2018) 34(06):706–7.

[30] Li H Wang YL Shang XM. Treatment of 61 cases of refractory heart failure after acute myocardial infarction with Qishen Ningxin prescription. Glob Trad Chin Med. (2023) 16(02):332–5. 10.3969/j.issn.1674-1749.2023.02.031

[31] Niu MH. Observation on efficacy of modified Guizhi decoction combined with recombinant human brain natriuretic peptide on refractory heart failure. Chin J Modern Drug Appl. (2021) 15(14):188–90. 10.14164/j.cnki.cn11-5581/r.2021.14.072

[32] Peng XQ Liang FM Tan ZQ Chen CH. Discussion on clinical effect of Zhenwu decoction combined with dopamine and furosemide in the treatment of refractory heart failure. Chin J Modern Drug Appl. (2019) 13(15):136–8. 10.14164/j.cnki.cn11-5581/r.2019.15.077

[33] Du XY Liu N Li C. Observation on the curative effect of Shengmaiyixin decoction in the treatment of refractory heart failure of Qi and Yin deficiency and blood stasis type. Hubei J Trad Chin Med. (2018) 40(10):14–6.

[34] Wang XS Ding XL. Clinical study of Sanshen Guiqi mixture in the treatment of chronic refractory heart failure and intervention of HCY and cTnI. Guizhou Med J. (2022) 46(08):1291–2.

[35] Shang YM. Clinical observation on treatment of refractory heart failure with decoction of heart failure combined with western medicine. China Contin Med Educ. (2018) 10(17):142–4. 10.3969/j.issn.1674-9308.2018.17.070

[36] Wang MJ Shen JP. Clinical efficacy of Qiangxin Liniao recipe combined with tolvaptan in the treatment of refractory heart failure. Cardiovasc Dis Electron J Integr Tradit Chin West Med. (2020) 8(01):56 +60. 10.16282/j.cnki.cn11-9336/r.2020.01.044

[37] Li Y Chen HY Sha X. Study on the therapeutic effect of Huoxueyixin decoction combined with spironolactone on refractory heart failure caused by pulmonary heart disease. J Chengde Med Univ. (2023) 40(02):127–9. 10.15921/j.cnki.cyxb.2023.02.018

[38] Ye J Weng LX. Clinical effect analysis of integrated traditional Chinese and western medicine in treating chronic pulmonary heart disease complicated with refractory heart failure. China Foreign Med Treat. (2019) 38(26):170–2. 10.16662/j.cnki.1674-0742.2019.26.170

[39] Wang JR Liu QL Feng L Song WJ Xi HC Ren QCM. Effect of Qiliqiangxin capsules combined with levosimendan on cardiac function, NT-proBNP and blood lipid in patients with refractory heart failure: a retrospective cohort study. World J Integr Tradit West Med. (2020) 15(01):143–6. 10.13935/j.cnki.sjzx.200131

[40] Zhang J. *Clinical observation of Yangxin Huoxue Tongmai ointment on patients with refractory heart failure* (master’s thesis). Hunan University of Chinese Medicine (2022).

[41] Huang XY Qu WL Xiao J. Clinical effect observation of Yixinli capsule in the treatment of refractory heart failure. China Pract Med. (2022) 17(15):166–8. 10.14163/j.cnki.11-5547/r.2022.15.054

[42] Jiang WD Jiang CY. Clinical study on treating refractory heart failure with self-prepared Lixin granule combined with rh BNP. Chin J Integr Med Cardio Cerebrovasc Dis. (2017) 15(09):1065–7. 10.3969/j.issn.1672-1349.2017.09.015

[43] Ge LY Chen AL Hu JG. Clinical efficacy and safety of Yangxin Shengmai granules combined with recombinant human brain natriuretic peptide in the treatment of patients with refractory heart failure. Chin J Integr Med Cardio Cerebrovasc Dis. (2020) 18(18):3035–8. 10.12102/j.issn.1672-1349.2020.18.023

[44] Wang JQ. Observation on the therapeutic effect of ivabradine combined with Qiliqiangxin capsules in the treatment of refractory heart failure. Prev Treat Cardiovasc Dis. (2019) 02:51–2.

[45] Liu T Ma LP. Observation on curative effect of Qiliqiangxin capsule combined with Xinmailong injection in treatment of refractory heart failure. World Latest Med Inform. (2019) 19(52):248. 10.19613/j.cnki.1671-3141.2019.52.172

[46] Chen W Shu WJ. Effect of Shenfu Qiangxin pill combined with levosimendan on refractory heart failure and its influence on Nt-proBNP, cTnT and CK-MB. J Bethune Med Sci. (2019) 17(05):486–8. 10.16485/j.issn.2095-7858.2019.05.032

[47] Liu HB Niu L. Clinical study on Shenfu Qiangxin pills combined with levosimendan in treatment of refractory heart failure. Drugs Clin. (2019) 34(04):988–92. 10.7501/j.issn.1674-5515.2019.04.022

[48] Zhao YL. Application study of integrative medicine in treatment of refractory heart failure. China J Modern Med. (2017) 27(19):91–5. 10.3969/j.issn.1005-8982.2017.19.019

[49] Li H Wang YL Shang XM. Clinical study on Quanxinkang capsules combined with ivabradine in treatment of refractory heart failure. Drugs Clin. (2023) 38(04):849–52. 10.7501/j.issn.1674-5515.2023.04.015

[50] Xu J Zhou X Zhao C Li XL. Efficacy analysis of Zuoximengdan combined with Qiliqiangxin capsules on refractory heart failure. China Foreign Med Treat. (2021) 40(32):95–7. 10.16662/j.cnki.1674-0742.2021.32.095

[51] Xing SC. *The clinical effect observation of Shenfu injection combined with continuous hemofiltration in the treatment of patients with refractory heart failure* (master’s thesis). School of Basic Medical ScienceXinxiang Medical University (2017).

[52] Ma XY. Effect of *Astragalus membranaceus* combined with dobutamine and phentolamine in the treatment of chronic pulmonary heart disease with refractory heart failure. J Xinxiang Med Univ. (2021) 38(02):133–6. 10.7683/xxyxyxb.2021.02.007

[53] Tan XQ Liu FJ. Clinical effect analysis of nitroglycerin and dopamine combined with Shengmai injection in treating refractory heart failure patients with pulmonary heart disease. Clin J Chin Med. (2016) 8(24):43–4. 10.3969/j.issn.1674-7860.2016.24.020

[54] Wang L Tian X Li BY. Effect of Xinmailong injection on cardiac function parameters and serum markers in elderly patients with refractory heart failure. Cardio Cerebrovasc Dis Prev Treat. (2020) 20(04):406–8. 10.3969/j.issn.1009-816x.2020.04.021

[55] Fan Y Zhang M Zheng JJ Gao X Zhu WW. Efficacy observation of Sofre injection combined with isosorbide mononitrate in the treatment of refractory heart failure. China Pharm. (2017) 20(12):2190–2.

[56] Li Q. Clinical analysis of Shengmai injection combined with dopamine in the treatment of refractory heart failure. World Latest Med Inform. (2016) 16(84):132. 10.3969/j.issn.1671-3141.2016.84.119

[57] Wang P. Effect of Shenmai injection combined with levosimendan in the treatment of dilated cardiomyopathy with refractory heart failure. Chin Gen Pract. (2017) 20(S3):379–81.

[58] Zhang Y Yuan LS Liu Y J Hu XX Zheng Q. Effect of Xinmailong injection on endothelial function and soluble St2 level in patients with chronic pulmonary heart disease complicated with refractory heart failure. Chin J Gerontol. (2019) 39(17):4119–22. 10.3969/j.issn.1005-9202.2019.17.003

[59] Li HW Guan AP Liu SS. Observation on efficacy of Yiqifumai injection combined with western medicine in patients with refractory end-stage heart failure. Shaanxi J Tradit Chin Med. (2016) 37(08):989–90. 10.3969/j.issn.1000-7369.2016.08.023

[60] Tong M Fan H Gao Y Xie DW Wang XF. Clinical research on Xinmailong injection and Qihong capsule in the treatment of refractory heart failure. Chin J Integr Med Cardio Cerebrovasc Dis. (2016) 14(16):1825–8. 10.3969/j.issn.1672-1349.2016.16.001

[61] Cao JD Gao L He HB Liu HL Wang Q Chen L. Clinical observation of Shenfu injection combined with dapagliflozin in the treatment of 31 cases of refractory heart failure. Drug Eval. (2022) 19(10):598–602. 10.19939/j.cnki.1672-2809.2022.10.07

[62] Cao JD Liu QL Yuan XY Hu J Han YT Cao Y Efficacy of Shenfu injection combined with sacubitril valsartan sodium in treating patients with refractory heart failure. Jiangsu Med J. (2020) 46(07):701–5. 10.19460/j.cnki.0253-3685.2020.07.014

[63] Wang K Sun YM. Clinical effect of Shenfu injection combined with sacubitril and valsartan sodium in the treatment of refractory heart failure. Curr Med Res Pract. (2022) 7(23):72–4. 10.19347/j.cnki.2096-1413.202223020

[64] Zhao TY. Efficacy of Shenmai injection combined with levosimendan in the treatment of dilated cardiomyopathy refractory heart failure and its effect on cardiac function and BNP level in patients with dilated cardiomyopathy refractory heart failure. J North Pharm. (2017) 14(11):135–6.

[65] Chang WN Wang X Chu PP Ma KY. Clinical efficacy and influence on myocardial fibrosis of high-dose *Astragalus* injection combined with ivabradine in the refractory heart failure patients. J North China Univ Sci Technol Health Sci Ed. (2022) 24(05):388–92. 10.19539/j.cnki.2095-2694.2022.05.009

[66] He JZ Lu F Qin G Wu ZJ Hu QY. Therapeutic effect of Shenmai injection combined with milrinone in treatment of refractory heart failure. Chin Arch Tradit Chin Med. (2020) 38(01):203–6. 10.13193/j.issn.1673-7717.2020.01.049

[67] Zhang XW Fang D Zhao CL Wei YQ. Effect of Danshen Chuanxiongqin injection on heart and kidney function improvement in elderly patients with refractory heart failure and hypotension. Guangxi Med J. (2018) 40(02):158–61. 10.11675/j.issn.0253-4304.2018.02.12

[68] Zhang XH. Treatment effect of Shenqi injection in patients with refractory heart failure and its influence on myocardial fibrosis. J Hainan Med Univ. (2017) 23(10):1322–5. 10.13210/j.cnki.jhmu.20170503.005

[69] Yang K Sun L Dong L Yang JL Li N. Effects of Xinmailong injection on patients with pulmonary heart disease complicated by refractory heart failure and copeptin. Hebei Med J. (2019) 41(05):759–61. 10.3969/j.issn.1002-7386.2019.05.031

[70] Wu SW Zhang YE Fan QX. Effect of adjuvant Shenqi injection therapy on cardiac function and myocardial fibrosis in patients with refractory heart failure. J Hainan Med Univ. (2018) 24(04):456–9. 10.13210/j.cnki.jhmu.20180129.003

[71] Liang B Yan C Zhang L Yang Z Wang L Xian S The effect of acupuncture and moxibustion on heart function in heart failure patients: a systematic review and meta-analysis. Evid Based Complement Alternat Med. (2019) 2019:6074967. 10.1155/2019/6074967 31772597 PMC6854931

[72] Hui J Wang Y Zhao J Cong W Xu F. Effects of Tai chi on health status in adults with chronic heart failure: a systematic review and meta-analysis. Front Cardiovasc Med. (2022) 9:953657. 10.3389/fcvm.2022.953657 36158796 PMC9500215

[73] Taylor-Piliae R Finley BA. Benefits of Tai chi exercise among adults with chronic heart failure: a systematic review and meta-analysis. J Cardiovasc Nurs. (2020) 35(5):423–34. 10.1097/JCN.0000000000000703 32544110

[74] Mei B Yuan L Shu Y. Quantitative evidence of the effect of Baduanjin exercise on quality of life and cardiac function in adults with chronic heart failure. Complement Ther Clin Pract. (2023) 53:101775. 10.1016/j.ctcp.2023.101775 37717550

[75] Lai Q Zhang J. Effects of traditional Chinese eight brocade exercise with same frequency and different durations on the quality of life, 6-min walk and brain natriuretic peptide in patients with chronic heart failure. Exp Gerontol. (2023) 172:112059. 10.1016/j.exger.2022.112059 36526096

[76] Huang PN Yang HF Liu SL Chu QM Wang S Zhou XX Clinical research of Huiyang Yixin ointment in treating chronic heart failure by acupoint application. Chin J Integr Med Cardio Cerebrovasc Dis. (2022) 20(06):967–70. 10.12102/j.issn.1672-1349.2022.06.002

[77] Shah AM Mann DL. In search of new therapeutic targets and strategies for heart failure: recent advances in basic science. Lancet. (2011) 378(9792):704–12. 10.1016/S0140-6736(11)60894-5 21856484 PMC3486638

[78] Lachowska K Gruchala M Narkiewicz K Hering D. Sympathetic activation in chronic heart failure: potential benefits of interventional therapies. Curr Hypertens Rep. (2016) 18(7):51. 10.1007/s11906-016-0660-7 27193773

[79] Mollace V Gliozzi M Capuano A Rossi F. Modulation of RAAS-natriuretic peptides in the treatment of HF: old guys and newcomers. Int J Cardiol. (2017) 226:126–31. 10.1016/j.ijcard.2016.03.085 27075034

[80] Streby KA Shah N Ranalli MA Kunkler A Cripe TP. Nothing but net: a review of norepinephrine transporter expression and efficacy of ^131^I-mIBG therapy. Pediatr Blood Cancer. (2015) 62(1):5–11. 10.1002/pbc.25200 25175627 PMC4237663

[81] Kreusser MM Lehmann LH Haass M Buss SJ Katus HA Lossnitzer D. Depletion of cardiac catecholamine stores impairs cardiac norepinephrine re-uptake by downregulation of the norepinephrine transporter. PLoS One. (2017) 12(3):e0172070. 10.1371/journal.pone.0172070 28282374 PMC5345760

[82] Wang R Yan XM Wang Y Liu R Yuan HX Pei MR. Effect of Fuzi decoction based on chronic heart failure rats model and its mechanism. Chin Arch Tradit Chin Med. (2019) 37(04):788–92. 10.13193/j.issn.1673-7717.2019.04.004

[83] Dong ZY Fang YW He DF Jia CJ. Effects of modified Zhenwu decoction on cardiac function in rats with chronic heart failure. Modern J Integr Tradit Chin West Med. (2013) 22(17):1842–4.

[84] Liu C Li WJ Xie J. Influence of Zhenwu decoction on Ang and ALD in serum of heart failure rats. Chin Arch Tradit Chin Med. (2015) 33(06):1374–6. 10.13193/j.issn.1673-7717.2015.06.030

[85] Zhang JM Shang YD Wu XR Fu YJ Xie CY. Clinical efficacy of Xinmailong injection in the treatment of chronic heart failure: a meta analysis. Chin Gen Pract. (2014) 17(12):1388–93. 10.3969/j.issn.1007-9572.2014.12.016

[86] Hanna A Frangogiannis NG. Inflammatory cytokines and chemokines as therapeutic targets in heart failure. Cardiovasc Drugs Ther. (2020) 34(6):849–63. 10.1007/s10557-020-07071-0 32902739 PMC7479403

[87] Dick SA Epelman S. Chronic heart failure and inflammation: what do we really know? Circ Res. (2016) 119(1):159–76. 10.1161/CIRCRESAHA.116.308030 27340274

[88] Zhou B Wang DD Qiu Y Airhart S Liu Y Stempien-Otero A Boosting NAD level suppresses inflammatory activation of PBMCs in heart failure. J Clin Invest. (2020) 130(11):6054–63. 10.1172/JCI138538 32790648 PMC7598081

[89] Stanciu AE Stanciu MM Vatasescu RG. NT-proBNP and CA 125 levels are associated with increased pro-inflammatory cytokines in coronary sinus serum of patients with chronic heart failure. Cytokine. (2018) 111:13–9. 10.1016/j.cyto.2018.07.037 30098475

[90] Van Tassell BW Trankle CR Canada JM Carbone S Buckley L Kadariya D IL-1 blockade in patients with heart failure with preserved ejection fraction. Circ Heart Fail. (2018) 11(8):e005036. 10.1161/CIRCHEARTFAILURE.118.005036 30354558 PMC6545106

[91] Gullestad L Ueland T Vinge LE Finsen A Yndestad A Aukrust P. Inflammatory cytokines in heart failure: mediators and markers. Cardiology. (2012) 122(1):23–35. 10.1159/000338166 22699305

[92] Zhang X Li M Wang H. Astragaloside IV alleviates the myocardial damage induced by lipopolysaccharide via the toll-like receptor 4 (TLR4)/nuclear factor kappa B (Nf-κB)/proliferator-activated receptor alpha (PPARα) signaling pathway. Med Sci Monit. (2019) 25:7158–68. 10.12659/MSM.916030 31545785 PMC6775796

[93] Yan P Mao W Jin L Fang M Liu X Lang J Crude Radix Aconiti Lateralis Preparata (Fuzi) with *Glycyrrhiza* reduces inflammation and ventricular remodeling in mice through the TLR4/NF-κB pathway. Mediators Inflamm. (2020) 2020:5270508. 10.1155/2020/5270508 33132755 PMC7593747

[94] Li C Wang Y Qiu Q Shi T Wu Y Han J Qishenyiqi protects ligation-induced left ventricular remodeling by attenuating inflammation and fibrosis via STAT3 and Nf-κB signaling pathway. PLoS One. (2014) 9(8):e104255. 10.1371/journal.pone.0104255 25122164 PMC4133204

[95] Hong LL Zhang S Wang Q Zhao YT Zhu Q Peng C Effect of Zhenwu decoction on chronic heart failure rats based on RAAS/NF-κB/inflammatory factor cascade reaction. Chin Tradit Herbal Drugs. (2020) 51(05):1279–86. 10.7501/j.issn.0253-2670.2020.05.026

[96] Zarkovic N. Roles and functions of ROS and RNS in cellular physiology and pathology. Cells. (2020) 9(3):767. 10.3390/cells9030767 32245147 PMC7140712

[97] Song LL Xue YT. Oxidative stress in heart failure and the research progress in traditional Chinese medicine. World Chin Med. (2022) 17(12):1769–72. 10.3969/j.issn.1673-7202.2022.12.023

[98] Chen YY Li Q Pan CS Yan L Fan JY He K Qishenyiqi pills, a compound in Chinese medicine, protects against pressure overload-induced cardiac hypertrophy through a multicomponent and multi-target mode. Sci Rep UK. (2015) 5:11802. 10.1038/srep11802 PMC4488877 26136154

[99] Yu SY Qian H Tian DW Yang MM Li DF Xu H Linggui Zhugan decoction activates the SIRT1-AMPK-PGC1 alpha signaling pathway to improve mitochondrial and oxidative damage in rats with chronic heart failure caused by myocardial infarction. Front Pharmacol. (2023) 14:1074837. 10.3389/fphar.2023.1074837 PMC10113531 37089931

[100] Wang X Tang TJ Zhai MT Ge RR Wang L Huang JL Ling-Gui-Zhu-Gan decoction protects H9c2 cells against H_2_O_2_-induced oxidative injury via regulation of the Nrf2/Keap1/Ho-1 signaling pathway. Evid Based Compl Alt. (2020) 2020:8860603. 10.1155/2020/8860603 PMC7721500 33312223

[101] Zhao QF Liu H Wang XY Hou YL Liu KJ Wang HT. Qiliqiangxin capsule can delay the mitochondrial pathway apoptosis of myocardial cells injured by oxidative stress through Pi3K/AKT/GSK3β signaling pathway. Chin J Pharmacol Toxicol. (2019) 33(09):681.

[102] Song LJ Kong HL Jiang YK Huang HT. Ginsenoside-Rb1 improves autophagy ability of hypoxia cardiomyocytes of neonatal rats by adenosinemonophosphate-activated protein kinase pathway. China Med. (2019) 14(09):1425–9. 10.3760/j.issn.1673-4777.2019.09.035

[103] Kang PM Izumo S. Apoptosis and heart failure: a critical review of the literature. Circ Res. (2000) 86(11):1107–13. 10.1161/01.res.86.11.1107 10850960

[104] Wang Q Wu J Zeng Y Chen K Wang C Yang S Pyroptosis: a pro-inflammatory type of cell death in cardiovascular disease. Clin Chim Acta. (2020) 510:62–72. 10.1016/j.cca.2020.06.044 32622968

[105] Wu J Dong E Zhang Y Xiao H. The role of the inflammasome in heart failure. Front Physiol. (2021) 12:709703. 10.3389/fphys.2021.709703 34776995 PMC8581560

[106] Mishra PK Adameova A Hill JA Baines CP Kang PM Downey JM Guidelines for evaluating myocardial cell death. Am J Physiol Heart Circ Physiol. (2019) 317(5):H891–922. 10.1152/ajpheart.00259.2019 31418596 PMC6879915

[107] Lei W Zi W Ling Y Di H Nan L Xu L. Shenfuqiangxin capsule inhibits apoptosis through mitogen-activated protein kinase signal pathway in rats with cardio-renal syndrome induced by infrarenal aortic-clamping. J Tradit Chin Med. (2017) 37(1):80–7. 10.1016/S0254-6272(17)30030-4 29957913

[108] Wang Z Hao D Lv N Wang L. Effects of Shenfu Qiangxin pill on Bcl-2/Bax apoptosis in rats with cardiac and renal syndrome. J Basic Chin Med. (2016) 22(12):1616–9. 10.19945/j.cnki.issn.1006-3250.2016.12.015

[109] Zhan TW Guo SW Chen X Mo HR Wang H Feng PF Effects of Yiqi Huoxue formula on apoptosis of myocardial cells in rats with myocardial infarction. Glob Tradit Chin Med. (2020) 13(04):540–5. 10.3969/j.issn.1674-1749.2020.04.002

[110] Chen Y Tang Y Xiang Y Xie YQ Huang XH Zhang YC. Shengmai injection improved doxorubicin-induced cardiomyopathy by alleviating myocardial endoplasmic reticulum stress and caspase-12 dependent apoptosis. Biomed Res Int. (2015) 2015:952671. 10.1155/2015/952671 25839043 PMC4369903

[111] Sun BX Li XJ Cai QL Jia YJ. Effect of new Shengmai decoction on apoptosis and protein expression of Bax, Bcl-2, and Caspase-3 of cardiomyocyte induced by adriamycin. Chin Tradit Herbal Drugs. (2020) 51(02):433–8. 10.7501/j.issn.0253-2670.2020.02.021

[112] Fan XY Liao XQ Huang SM Wang ZY Li L Hu ZX. Shenfu injection improves chronic heart failure by regulating pyroptosis based on NLRP3/caspase-1 pathway. Zhongguo Zhong Yao Za Zhi. (2023) 48(23):6475–82. 10.19540/j.cnki.cjcmm.20230914.701 38212004

[113] Yan SH Wang H Pan JL Yang HM. Effects of Shenkui Tongmai granule on the charred death of myocardial cells and related factors in rats with chronic heart failure. J Basic Chin Med. (2019) 25(02):168–70. 10.19945/j.cnki.issn.1006-3250.2019.02.013

[114] Binder P Wang S Radu M Zin M Collins L Khan S Pak2 as a novel therapeutic target for cardioprotective endoplasmic reticulum stress response. Circ Res. (2019) 124(5):696–711. 10.1161/CIRCRESAHA.118.312829 30620686 PMC6407830

[115] Lou LX Wu AM Zhang DM Wu SX Gao YH Nie B Yiqi Huoxue recipe improves heart function through inhibiting apoptosis related to endoplasmic reticulum stress in myocardial infarction model of rats. Evid Based Complement Alternat Med. (2014) 2014:745919. 10.1155/2014/745919 24864159 PMC4016842

[116] Li L Zhao Q Kong W. Extracellular matrix remodeling and cardiac fibrosis. Matrix Biol. (2018) 68–69:490–506. 10.1016/j.matbio.2018.01.013 29371055

[117] Zhen YZ Song SH Xing J. Qili Qiangxin capsule combined with topavtan tablets in regulation of MMPs/TIMPs of patients with coronary heart disease chronic heart failure. Chin Arch Tradit Chin Med. (2021) 39(09):165–8. 10.13193/j.issn.1673-7717.2021.09.041

[118] Cheng S Yu P Yang L Shi H He A Chen H Astragaloside IV enhances cardioprotection of remote ischemic conditioning after acute myocardial infarction in rats. Am J Transl Res. (2016) 8(11):4657–69. 27904669 PMC5126311

[119] Jiang HQ Zhang JG Tan HY Wei XQ. Effects of astragaloside iv on myocardial fibrosis and connective tissue growth factor expression in experimental rats with chronic heart failure. Chin Circ J. (2016) 31(02):165–9. 10.3969/j.issn.1000-3614.2016.02.015

[120] Li YP Ruan XF Xu XW Qiang TT Li W Wang XL. Effect of Shengmai injection on Ang II-induced cardiomyocyte hypertrophy andapoptosis and its effect on energy metabolism. Liaoning J TraditChin Med. (2020) 47(09):18–22. 10.13192/j.issn.1000-1719.2020.09.006

[121] Wei XH Guo X Pan CS Li H Cui YC Yan L Quantitative proteomics reveal that metabolic improvement contributes to the cardioprotective effect of T89 on isoproterenol-induced cardiac injury. Front Physiol. (2021) 12:653349. 10.3389/fphys.2021.653349 34262469 PMC8273540

[122] Han A Lu Y Zheng Q Zhang J Zhao Y Zhao M Qiliqiangxin attenuates cardiac remodeling via inhibition of TGF-Beta1/Smad3 and Nf-κB signaling pathways in a rat model of myocardial infarction. Cell Physiol Biochem. (2018) 45(5):1797–806. 10.1159/000487871 29510381

[123] Wang YX Zhu MJ Zhu XF Li B. Effect of Shenfu Yixin granule on heart failure rats myocardial C-fos, C-myc expression. Chin J Exp Tradit Med Formulae. (2011) 17(03):145–7. 10.13422/j.cnki.syfjx.2011.03.051

[124] Uthman L Homayr A Juni RP Spin EL Kerindongo R Boomsma M Empagliflozin and dapagliflozin reduce ROS generation and restore NO bioavailability in tumor necrosis factor α-stimulated human coronary arterial endothelial cells. Cell Physiol Biochem. (2019) 53(5):865–86. 10.33594/000000178 31724838

[125] Zhang Q Liu J Duan H Li R Peng W Wu C. Activation of Nrf2/HO-1 signaling: an important molecular mechanism of herbal medicine in the treatment of atherosclerosis via the protection of vascular endothelial cells from oxidative stress. J Adv Res. (2021) 34:43–63. 10.1016/j.jare.2021.06.023 35024180 PMC8655139

[126] Gutierrez E Flammer AJ Lerman LO Elizaga J Lerman A Fernandez-Aviles F. Endothelial dysfunction over the course of coronary artery disease. Eur Heart J. (2013) 34(41):3175–81. 10.1093/eurheartj/eht351 24014385 PMC3814514

[127] Wang H Wu J Wei GL. Effects of Shensong Yangxin capsule and metoprolol on vascular endothelial function, vascular elasticity and major adverse cardiovascular events in patients with coronary heart disease. Curr Med Res Pract. (2022) 7(31):154–7. 10.19347/j.cnki.2096-1413.202231042

[128] Zhang ZM Huang YM. Effect of Qili Qiangxin capsules on myocardial injury and vascular endothelial function in patients after PCI. New Chin Med. (2021) 53(14):93–6. 10.13457/j.cnki.jncm.2021.14.025

[129] Zhu J Song W Xu S Ma Y Wei B Wang H Shenfu injection promotes vasodilation by enhancing eNOS activity through the PI3K/Akt signaling pathway in vitro. Front Pharmacol. (2020) 11:121. 10.3389/fphar.2020.00121 32161546 PMC7054240

[130] He Y Huang W Zhang C Chen L Xu R Li N Energy metabolism disorders and potential therapeutic drugs in heart failure. Acta Pharm Sin B. (2021) 11(5):1098–116. 10.1016/j.apsb.2020.10.007 34094822 PMC8144890

[131] van Bilsen M Smeets PJ Gilde AJ van der Vusse GJ. Metabolic remodelling of the failing heart: the cardiac burn-out syndrome? Cardiovasc Res. (2004) 61(2):218–26. 10.1016/j.cardiores.2003.11.014 14736538

[132] Martin-Fernandez B Gredilla R. Mitochondria and oxidative stress in heart aging. Age (Dordr). (2016) 38(4):225–38. 10.1007/s11357-016-9933-y 27449187 PMC5061683

[133] Zhou F Fang W Zhu GL. Shenfu Qiangxin mixture effects on AMPK-PGC-1 pathway of myocardial energy metabolism in heart failure rats. Zhejiang J Integr Tradit Chin West Med. (2018) 28(10):834–7.

[134] Huang Y Zhang K Jiang M Ni J Chen J Li L Regulation of energy metabolism by combination therapy attenuates cardiac metabolic remodeling in heart failure. Int J Biol Sci. (2020) 16(16):3133–48. 10.7150/ijbs.49520 33162820 PMC7645995

[135] Yu JH Li YH Liu XY Ma Z Michael S Orgah JO Mitochondrial dynamics modulation as a critical contribution for Shenmai injection in attenuating hypoxia/reoxygenation injury. J Ethnopharmacol. (2019) 237:9–19. 10.1016/j.jep.2019.03.033 30880258

[136] Han X Zhang Y Qiao O Ji H Zhang X Wang W Proteomic analysis reveals the protective effects of Yiqi Fumai lyophilized injection on chronic heart failure by improving myocardial energy metabolism. Front Pharmacol. (2021) 12:719532. 10.3389/fphar.2021.719532 34630097 PMC8494180

[137] Sun K Li YY Jin J. A double-edged sword of immuno-microenvironment in cardiac homeostasis and injury repair. Signal Transduct Target Ther. (2021) 6(1):79. 10.1038/s41392-020-00455-6 33612829 PMC7897720

[138] Winter MP Sulzgruber P Koller L Bartko P Goliasch G Niessner A. Immunomodulatory treatment for lymphocytic myocarditis—a systematic review and meta-analysis. Heart Fail Rev. (2018) 23(4):573–81. 10.1007/s10741-018-9709-9 29862463 PMC6010497

[139] Wang H Hu YH Song QQ Qiu ZL Bo RQ. The impact of Shenfu injection on the immune function in patients with chronic heart failure and heart kidney Yang deficiency syndrome. Chin J Integr Med Cardio Cerebrovasc Dis. (2016) 14(13):1441–5. 10.3969/j.issn.1672-1349.2016.13.001

[140] Zhang DC Zhang CY Lu MX. Effects of Qiliqiangxin capsule combined with conventional western medicines on ventricular remodeling and related immune indexes of patients with chronic congestive heart failure. Hebei Med J. (2017) 39(21):3209–12. 10.3969/j.issn.1002-7386.2017.21.002

[141] He X Ji YX. Effect of co-administration of Linggui Zhugan decoction and western medicine on inflammatory cytokines, and immune and cardiac functions of patients with chronic heart failure. Trop J Pharm Res. (2019) 18(2):365–70. 10.4314/tjpr.v18i2.20

[142] Liu YY Li H Zhu YS. Effects of Qishen Yiqi dropping pills on cardiac function, immune function and microRna155 level in patients with coronary heart disease and chronic heart failure. Chin J Integr Med Cardio Cerebrovasc Dis. (2017) 15(11):1342–4. 10.3969/j.issn.1672-1349.2017.11.015

[143] Shi SN. *Exploring the Immunoregulatory Mechanism of Shenqi Compound Recipe in the Treatment of Ischemic Heart Failure Based on IL-6/Stat3 Pathway* [doctor’s thesis]. China Academy of Chinese Medical Sciences (2022).

[144] Butter C Rastogi S Minden HH Meyhofer J Burkhoff D Sabbah HN. Cardiac contractility modulation electrical signals improve myocardial gene expression in patients with heart failure. J Am Coll Cardiol. (2008) 51(18):1784–9. 10.1016/j.jacc.2008.01.036 18452785

[145] Lompre AM Hajjar RJ Harding SE Kranias EG Lohse MJ Marks AR. Ca^2+^ cycling and new therapeutic approaches for heart failure. Circulation. (2010) 121(6):822–30. 10.1161/CIRCULATIONAHA.109.890954 20124124 PMC2834781

[146] Yu SX Yang YL Zuo ZF Lu ML Lv T Liu XZ. Role of calcium-sensing receptor in prevention and treatment ofcardiomyocyte injury with ginsenoside Rg1. Chin J Geriatr Heart Brain Vessel Dis. (2018) 20(07):752–6. 10.3969/j.issn.1009-0126.2018.07.019

[147] Lu M Wang H Wang J Zhang J Yang J Liang L Astragaloside IV protects against cardiac hypertrophy via inhibiting the Ca^2+^/CaN signaling pathway. Planta Med. (2014) 80(1):63–9. 10.1055/s-0033-1360129 24338553

[148] Qian W Xiong X Fang Z Lu H Wang Z. Protective effect of tetramethylpyrazine on myocardial ischemia-reperfusion injury. Evid Based Complement Alternat Med. (2014) 2014:107501. 10.1155/2014/107501 25152756 PMC4135172

[149] Yang XJ Chen XL. Influences of Qishen granules on cardiac function and calcium ionconcentration of myocardial cells in heart failure rats. Curr Med Res Pract. (2020) 5(13):1–3. 10.19347/j.cnki.2096-1413.202013001

[150] Lyu LF Wei XQ Wang MG Zhang XR Ma XZ Dorje D Effects of Wenyang Yiqi huoxue prescription on Na^+^-K^+^-ATPase and Ca^2+^-ATPase in myocardial tissue of rats with chronic heart failure. Chin J Inform Tradit Chin Med. (2023) 30(02):74–80. 10.19879/j.cnki.1005-5304.202205682

[151] Li B Chi RF Qin FZ Guo XF. Distinct changes of myocyte autophagy during myocardial hypertrophy and heart failure: association with oxidative stress. Exp Physiol. (2016) 101(8):1050–63. 10.1113/EP085586 27219474

[152] Ikeda S Zablocki D Sadoshima J. The role of autophagy in death of cardiomyocytes. J Mol Cell Cardiol. (2022) 165:1–8. 10.1016/j.yjmcc.2021.12.006 34919896 PMC8940676

[153] Riquelme JA Chavez MN Mondaca-Ruff D Bustamante M Vicencio JM Quest AF Therapeutic targeting of autophagy in myocardial infarction and heart failure. Expert Rev Cardiovasc Ther. (2016) 14(9):1007–19. 10.1080/14779072.2016.1202760 27308848

[154] Gao T Zhang SP Wang JF Liu L Wang Y Cao ZY TLR3 contributes to persistent autophagy and heart failure in mice after myocardial infarction. J Cell Mol Med. (2018) 22(1):395–408. 10.1111/jcmm.13328 28945004 PMC5742674

[155] Liao XQ Huang SM Fan XY Wang ZY Zhang Q Hu ZX. Protective effect of Shenfu injection on regulation of autophagy in rats with chronic heart failure based on PI3K/Akt/mTOR pathway. Zhongguo Zhong Yao Za Zhi. (2023) 48(21):5908–14. 10.19540/j.cnki.cjcmm.20230619.502 38114187

[156] Rub C Wilkening A Voos W. Mitochondrial quality control by the Pink1/Parkin system. Cell Tissue Res. (2017) 367(1):111–23. 10.1007/s00441-016-2485-8 27586587

[157] Yu W Gao B Li N Wang J Qiu C Zhang G Sirt3 deficiency exacerbates diabetic cardiac dysfunction: role of Foxo3A-Parkin-mediated mitophagy. Biochim Biophys Acta Mol Basis Dis (2017) 1863(8):1973–83. 10.1016/j.bbadis.2016.10.021 27794418

[158] Hsieh CC Li CY Hsu CH Chen HL Chen YH Liu YP Mitochondrial protection by simvastatin against angiotensin II-mediated heart failure. Br J Pharmacol. (2019) 176(19):3791–804. 10.1111/bph.14781 31265743 PMC6780047

[159] Yu Z Chen R Li M Yu Y Liang Y Han F Mitochondrial calcium uniporter inhibition provides cardioprotection in pressure overload-induced heart failure through autophagy enhancement. Int J Cardiol. (2018) 271:161–8. 10.1016/j.ijcard.2018.05.054 29803339

[160] Guan Z Chen J Wang L Hao M Dong X Luo T Nuanxinkang prevents the development of myocardial infarction-induced chronic heart failure by promoting Pink1/Parkin-mediated mitophagy. Phytomedicine. (2023) 108:154494. 10.1016/j.phymed.2022.154494 36279758

[161] Guan S Xin Y Ding Y Zhang Q Han W. Ginsenoside Rg1 protects against cardiac remodeling in heart failure via Sirt1/Pink1/Parkin-mediated mitophagy. Chem Biodivers. (2023) 20(2):e202200730. 10.1002/cbdv.202200730 36639922

[162] Morrow DA Velazquez EJ DeVore AD Desai AS Duffy CI Ambrosy AP Clinical outcomes in patients with acute decompensated heart failure randomly assigned to sacubitril/valsartan or enalapril in the PIONEER-HF trial. Circulation. (2019) 139(19):2285–8. 10.1161/CIRCULATIONAHA.118.039331 30955360

[163] McMurray JJ Packer M Desai AS Gong J Lefkowitz MP Rizkala AR Angiotensin-neprilysin inhibition versus enalapril in heart failure. N Engl J Med. (2014) 371(11):993–1004. 10.1056/NEJMoa1409077 25176015

[164] Comin-Colet J Manito N Segovia-Cubero J Delgado J Garcia Pinilla JM Almenar L Efficacy and safety of intermittent intravenous outpatient administration of levosimendan in patients with advanced heart failure: the LION-HEART multicentre randomised trial. Eur J Heart Fail. (2018) 20(7):1128–36. 10.1002/ejhf.1145 29405611

[165] Packer M Anker SD Butler J Filippatos G Pocock SJ Carson P Cardiovascular and renal outcomes with empagliflozin in heart failure. N Engl J Med. (2020) 383(15):1413–24. 10.1056/NEJMoa2022190 32865377

[166] Writing C Maddox TM Januzzi JL Jr Allen LA Breathett K Butler J 2021 update to the 2017 ACC expert consensus decision pathway for optimization of heart failure treatment: answers to 10 pivotal issues about heart failure with reduced ejection fraction: a report of the American College of Cardiology Solution Set Oversight Committee. J Am Coll Cardiol (2021) 77(6):772–810. 10.1016/j.jacc.2020.11.022 33446410

